# Advances in Diagnosis, Surveillance, and Monitoring of Zika Virus: An Update

**DOI:** 10.3389/fmicb.2017.02677

**Published:** 2018-01-19

**Authors:** Raj K. Singh, Kuldeep Dhama, Kumaragurubaran Karthik, Ruchi Tiwari, Rekha Khandia, Ashok Munjal, Hafiz M. N. Iqbal, Yashpal S. Malik, Rubén Bueno-Marí

**Affiliations:** ^1^ICAR-Indian Veterinary Research Institute, Bareilly, India; ^2^Division of Pathology, ICAR-Indian Veterinary Research Institute, Bareilly, India; ^3^Central University Laboratory, Tamil Nadu Veterinary and Animal Sciences University, Chennai, India; ^4^Department of Veterinary Microbiology and Immunology, College of Veterinary Sciences, UP Pandit Deen Dayal Upadhayay Pashu Chikitsa Vigyan Vishwavidyalay Evum Go-Anusandhan Sansthan, Mathura, India; ^5^Department of Biochemistry and Genetics, Barkatullah University, Bhopal, India; ^6^School of Engineering and Science, Tecnologico de Monterrey, Monterrey, Mexico; ^7^Division of Biological Standardization, ICAR-Indian Veterinary Research Institute, Bareilly, India; ^8^Laboratorios Lokímica, Departamento de Investigación y Desarrollo (I+D), Valencia, Spain

**Keywords:** Zika virus, Zika fever, diagnosis, surveillance, monitoring

## Abstract

Zika virus (ZIKV) is associated with numerous human health-related disorders, including fetal microcephaly, neurological signs, and autoimmune disorders such as Guillain-Barré syndrome (GBS). Perceiving the ZIKA associated losses, in 2016, the World Health Organization (WHO) declared it as a global public health emergency. In consequence, an upsurge in the research on ZIKV was seen around the globe, with significant attainments over developing several effective diagnostics, drugs, therapies, and vaccines countering this life-threatening virus at an early step. State-of-art tools developed led the researchers to explore virus at the molecular level, and in-depth epidemiological investigations to understand the reason for increased pathogenicity and different clinical manifestations. These days, ZIKV infection is diagnosed based on clinical manifestations, along with serological and molecular detection tools. As, isolation of ZIKV is a tedious task; molecular assays such as reverse transcription-polymerase chain reaction (RT-PCR), real-time qRT-PCR, loop-mediated isothermal amplification (LAMP), lateral flow assays (LFAs), biosensors, nucleic acid sequence-based amplification (NASBA) tests, strand invasion-based amplification tests and immune assays like enzyme-linked immunosorbent assay (ELISA) are in-use to ascertain the ZIKV infection or Zika fever. Herein, this review highlights the recent advances in the diagnosis, surveillance, and monitoring of ZIKV. These new insights gained from the recent advances can aid in the rapid and definitive detection of this virus and/or Zika fever. The summarized information will aid the strategies to design and adopt effective prevention and control strategies to counter this viral pathogen of great public health concern.

## Introduction

Zika virus (ZIKV) is the latest emergent virus after the Ebola epidemic. In the past, ZIKV has only been associated with mild disease; however, after subdue activity for six decades, it recently emerged as a significant threat to human health, with evident fetal abnormalities, microcephaly, serious neurological complications, and autoimmune disorders such as Guillain-Barré syndrome (GBS) (Al-Qahtani et al., 2016; Cao-Lormeau et al., 2016; Carteaux et al., 2016; Mlakar et al., 2016; Singh et al., 2016, 2017). ZIKV is a mosquito-borne virus in the genus *Flavivirus* of the *Flaviviridae* family that is transmitted by *Aedes aegypti* and *Ae. albopictus* mosquitoes, with the potential for rapid spread (Musso and Gubler, 2016; Weaver et al., 2016). ZIKV is an enveloped, single-stranded RNA virus that is closely related to dengue virus (DENV), yellow fever virus (YFV), and West Nile virus (WNV) (Fajardo et al., 2016; Kruger, 2016). The first isolation of ZIKV from the blood of a rhesus monkey from the Zika forest in Uganda was reported in 1947 (Hayes, 2009). There is increasing evidence of the placental transfer of ZIKV, as well as its ability to affect neuronal tissue of a growing fetus (Martines et al., 2016; Mlakar et al., 2016). Autoimmune complications like GBS and maternal transfer to fetuses resulting in microcephaly has become particularly alarming. A lack of suitable vaccines and drugs has complicated the response to the recent epidemic. The presently available treatment options remain supportive in nature. Hence, regular surveillance is advised, especially for travelers and pregnant women (Yadav et al., 2016). Furthermore, predisposing factors like climate change, globalization, an explosive population rise, and increased urbanization have aided the spread of this virus, posing pandemic potential (Haug et al., 2016; Troncoso, 2016).

Various platforms are available for ZIKV diagnosis. Isolation remains a difficult task, as it requires adequate biocontainment facilities to handle the samples. Hence, other assays like reverse transcription-polymerase chain reaction (RT-PCR) and enzyme-linked immunosorbent assay (ELISA) have been developed for ZIKV diagnosis (Landry and St. George, 2017; Rather et al., 2017). RT-PCR can be performed using urine, blood, and cerebrospinal fluid (CSF) from an early stage in infection. IgM and IgG ELISAs are available for diagnosis of ZIKV antibodies in serum (Shan et al., 2017a). Conventional diagnostics have the limitation of cross-reactivity, because ZIKV is genetically similar to other flaviviruses. Investigators have proposed several new diagnostic tools for early and accurate diagnosis of ZIKV infection (Miller et al., 2017; Sharma and Lal, 2017). Newer and faster techniques like biosensors, loop-mediated isothermal amplification (LAMP), and lateral flow assays (LFAs) have been developed recently, and they are being validated for commercialization (Sakudo et al., 2016). In addition, to spread awareness about ZIKV, people are sharing relevant photographs on popular social media platforms such as Pinterest (San Francisco, CA) and Instagram (Menlo Park, CA) as adjunct health educational tools (Fung et al., 2017). The objective of present review is to outline the recent technological progresses in designing and developing rapid and confirmatory diagnostic, surveillance, and monitoring approaches to counter ZIKV. This review brings up-to-date information on new achievements in the field of ZIKV diagnosis, and opines the core features of the in-use and forthcoming assays. The modern assays are speedy as well possess higher competence befitting the clinical diagnosis. Here, clinical to innovative means for rapid ZIKV detection and identification, precisely, clinical, gross and histopathological lesion-based diagnosis, and detection methods involving isolation of ZIKV, serological assays viz. multiplex microsphere immunoassay (MIA), Zika IgM antibody capture enzyme-linked immunosorbent assay (Zika MAC-ELISA), reporter virus neutralization test (RVNT), plaque reduction neutralization test and molecular assays like (RT)-PCR, real-time qRT-PCR, reverse transcription strand invasion based amplification (RT-SIBA) assay; nucleic acid sequence-based amplification (NASBA) coupled with CRISPR detection module, real-time loop-mediated isothermal amplification (RT-LAMP), transcription-mediated amplification (TMA) technology, reverse transcription isothermal recombinase polymerase amplification (RPA), flow cytometry, surface plasmon resonance-based technology, are reviewed and discussed.

### Trends and advances in diagnosis, surveillance, and monitoring of ZIKV

The clinical signs of Zika fever are not pathognomic, therefore, confirmation of ZIKV infection requires isolation and identification of the virus. Diagnosis of ZIKV infection can be made based on apparent pathology, presence of the vector in the area, ZIKV-associated neurological syndromes, epidemiological evidence, and results of serological and molecular tests (Singh et al., 2016). ZIKV has been shown to be transmitted through body fluids during sexual intercourse and vertically, from mother to fetus (Balm et al., 2012; Gourinat et al., 2015; Leung et al., 2015; Musso et al., 2015; Tognarelli et al., 2015; de M. Campos et al., 2016; Jacob, 2016; Moulin et al., 2016; Shinohara et al., 2016; Staples et al., 2016; Pyzocha et al., 2017). Clinical samples that can be used for diagnostic purposes comprises of serum, the umbilical cord of an infant, urine, nasopharyngeal swabs, saliva, brain tissue, amniotic fluid, CSF, and placenta. In infected infants, ZIKV can be identified by immunohistochemical staining of the antigen in the umbilical cord and placenta (Staples et al., 2016). Diagnosis of ZIKV-infected stillborn fetuses can be made by histopathological and immunohistochemical staining (Landry and St. George, 2017). As mentioned earlier, most in-practice laboratory diagnostic tools include RT-PCR, multiplex PCR, antibody-capture ELISA, sandwich ELISA, and indirect immunofluorescent test (IIFT) (Steinhagen et al., 2016; Miller et al., 2017). Nucleic acid detection methods have been found most reliable, quick, sensitive, specific, and economical. Other molecular and serological tests can be used to confirm ZIKV infection (Staples et al., 2016). Differential diagnosis is an essential part of successful ZIKV detection as most of the time they are confused with Dengue virus at the early part of infection. ZIKV has attracted interest among researchers only in the recent years hence due to lack of laboratories that can accurately diagnose ZIKV most of the occasions they are not diagnosed properly (Hamel et al., 2016; Shankar et al., 2017).

### Clinical, gross, and histopathological lesion-based diagnosis

Viruria has been detected for more than 15 days after the onset of symptoms (Rozé et al., 2016). The clinical signs of ZIKV infection like fever, headache, myalgia, arthralgia, and maculopapular rash are inconsistent, as only one out of 4–5 people exhibit them. Hence, diagnosis based on clinical signs may not be reliable (Eppes et al., 2017). ZIKV replicates in virus-induced membranous replication factories (RFs). Endoplasmic reticulum of ZIKV infected human hepatic cells and neural progenitor cells show invaginations with pore-like openings toward the cytosol. Upon electron microscopy, it was revealed that the infection with ZIKV cause drastic change in microtubules and intermediate filaments organization forming cage like structure. Also, cytoskeleton-targeting drugs affect ZIKV infection severely and tight linking of ZIKV RF with host cell cytoskeleton changes is indicative of infection (Cortese et al., 2017). Leukopenia and thrombocytopenia are less common, whereas peripheral edema and conjunctivitis are more commonly encountered in ZIKV infections (Ioos et al., 2014; Rafiei et al., 2016). Occipitofrontal circumference can indicate microcephaly. To detect microcephaly in pregnant women, ultrasound testing is used (Lazear and Diamond, 2016; Oliveira Melo et al., 2016; Society for Maternal-Fetal Medicine (SMFM) Publications Committee, 2016). Epidemiological data is collected from maternal-fetal obstetrician/gynecologists, pregnant patients, and Zika-suspected cases to determine the threat of congenital ZIKV infection. The various kinds of deformities are based on the time of ZIKV exposure and also on the replication of ZIKV in the fetal and placental tissues. These data-gatherer techniques overcome the limitations of ultrasound-based strategies, where diagnosis depends exclusively on the detection of microcephaly and offer an alternative to detect malformations (Eppes et al., 2017).

### Neuroimaging diagnosis

**Figure 1:**
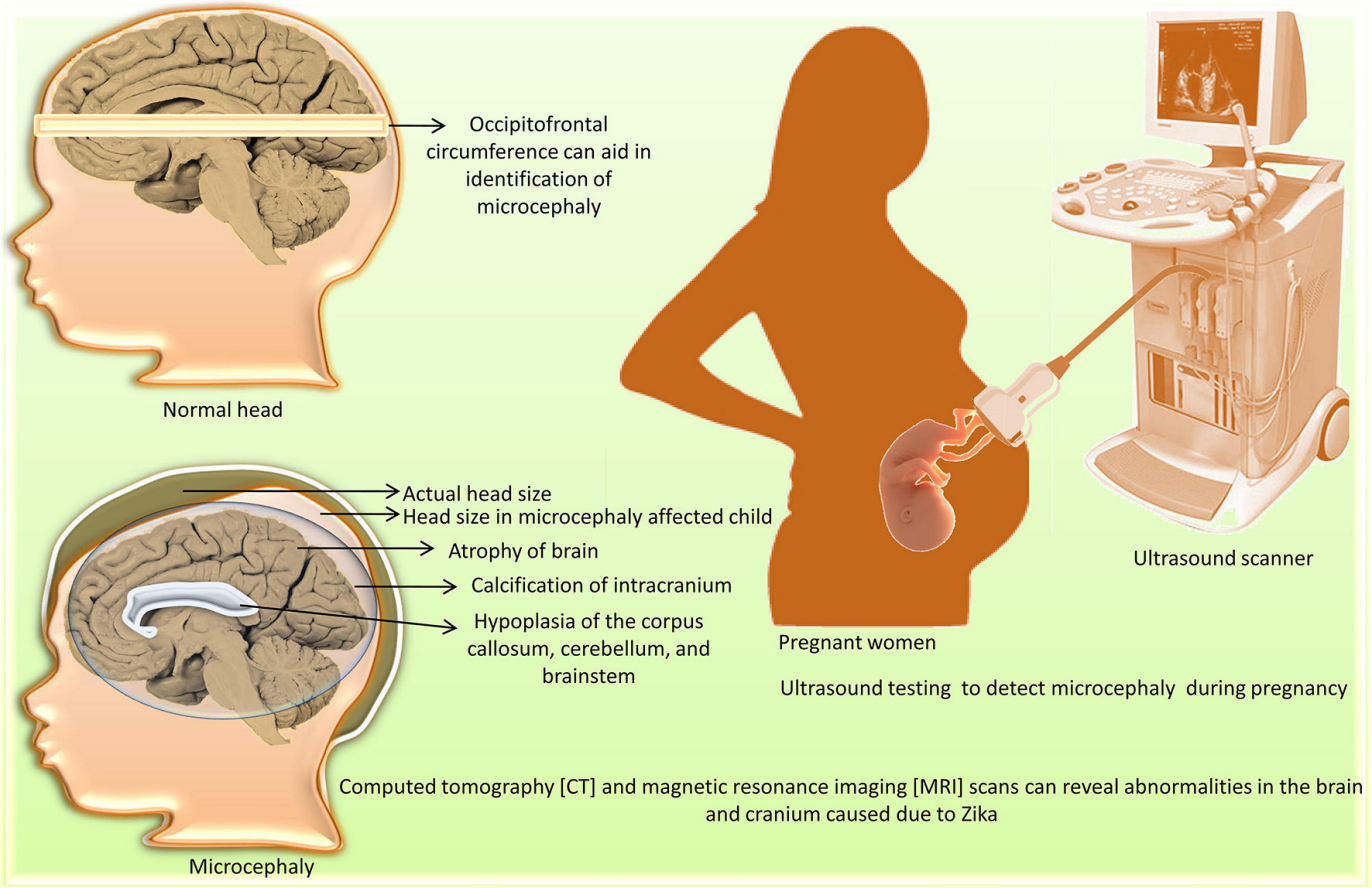
Diagnosis of microcephaly caused by Zika virus.

Neuroimaging [computed tomography (CT) and magnetic resonance imaging (MRI) scans] can reveal congenital microcephaly (de Fatima Vasco Aragao et al., 2016). Abnormalities include atrophy of the brain parenchyma with secondary ventriculomegaly; calcification of the intracranium; cortical development malformations such as polymicrogyria and lissencephaly-pachygyria; hypoplasia of the corpus callosum, cerebellum, and brainstem; and hearing loss (Figure 1). Other abnormalities associated with ZIKV infection, including ocular abnormalities and arthrogryposis, can be found in infected fetuses. Post-natal (acquired) ZIKV infection can result in either a mild symptomatic or asymptomatic course, whereas prenatal (congenital) ZIKV infection can be severe, leading to brain abnormalities known as congenital Zika syndrome (Zare Mehrjardi et al., 2016, 2017). Intra-uterine ZIKV infection is characterized by reduced cortical gyration and white-matter hypomyelination or dysmyelination with cerebellar hypoplasia in majority of fetuses and newborns (Araujo Júnior et al., 2017). A marked ZIKV associated feature is abnormal head shape. Skull gains a collapsed shape with everted and/or cupped sutures with overriding bones in the occipital region leading to formation of redundant and folded skin. Calcified skull also needs to be taken into account for post-natal assessment (de Oliveira-Szejnfeld et al., 2017). A recent neuroimaging study of 17 fetuses with confirmed ZIKV showed that microcephaly occur in fetus at a minimum of 15 weeks time period requirement after a pregnant woman gets infected with ZIKV (Parra-Saavedra et al., 2017).

### Isolation of ZIKV

ZIKV from a mosquito can be isolated in newborn Swiss albino mice via intraperitoneal, intracerebral, and subcutaneous inoculation (Marchette et al., 1969; Way et al., 1976). Hemagglutination inhibition test can be used to confirm virus in brain-passaged material (Haddow et al., 1964). For *in vitro* cultivation of the virus, Vero cells, rhesus monkey kidney cells (LLC- 192 MK2), and mosquito-origin [*A. albopictus* (C6/36), *A. psuedoscutellaris* (MOS61 or AP-61)] cell lines are employed (Barzon et al., 2016; Waggoner and Pinsky, 2016). Virus isolates from urine and saliva samples were recovered in Vero cell lines in one study, showing that Vero cell lines can be used to isolate ZIKV (Bonaldo et al., 2016). Another comparative study revealed that culture of ZIKV in C6/36 and Vero cells yield higher virus titers (Coelho et al., 2017). BHK21 suspension cell lines were also studied for the propagation of ZIKV to aid in ZIKV vaccine production (Nikolay et al., in press). Because live ZIKV sample handling requires specialized facilities to prevent its spread to handlers, not all laboratories can isolate the virus. Low-level viremias can also limit the opportunity for isolation of the virus (Lanciotti et al., 2008; Zanluca and Dos Santos, 2016). To test vaccines, several laboratory animal models of ZIKV infection have been evaluated. Two mouse models were studied, namely A129 type I interferon receptor knockout mice and AG129 type I and type II interferon receptor knockout mice. Three-week-old A129 and AG129 mice showed neurological signs and the presence of virus in their brains 7 days after challenge, whereas immunocompetent mice did not produce similar signs when challenged with ZIKV (Brault and Bowen, 2016). In one study, 15 non-human and 18 human cell lines were used to determine the replication of ZIKV in various cells. Results revealed that ZIKV replicated in neuronal (SF268), retinal (ARPE19), pulmonary (Hep-2 and HFL), hepatic (Huh-7), placental (JEG-3), muscle (RD), and colonic (Caco-2) cell lines. Replication in placental cell lines shows the ability of ZIKV to cross the placental barrier. ZIKV replication was also found in non-human cell lines, namely pig (PK-15), chicken (DF-1), non-human primate (Vero and LLC-MK2), rabbit (RK-13), and hamster (BHK21) cell lines; hence, these animals have been suggested as animal models for ZIKV (Chan J. F.-W et al., 2016).

### Serological tests

Hemagglutination inhibition, serum neutralization, and complement fixation are the useful tests for the diagnosis of Zika fever (Fagbami, 1979; Monath et al., 1980). Short viremias make it difficult to detect ZIKV; hence, serological diagnosis is a better way out to determine the status of an individual for a longer period of time (Shan et al., 2017a). Mouse protection test has also been employed to identify ZIKV antibodies in serum samples (Kirya and Okia, 1977). Monitoring of IgG and IgM antibodies in serum by ELISA is highly useful for serological diagnosis of ZIKV (Staples et al., 2016). Seroconversion is confirmed by analyzing the antibodies titers in paired serum samples (acute and convalescent phase) during the course of infection. Recently neutralization assay was combined with real time PCR platform to detect the end point so that there was 100% sensitivity and specificity of detection of ZIKV. Authors suggest this assay can be a confirmatory test for the samples positive by commercial immunoassays for detection ZIKV antibodies (Wilson et al., 2017).

#### Plaque reduction neutralization test

Plaque reduction neutralization test (PRNT) is used to detect antibodies against ZIKV and shows less cross-reactivity with antibodies against other *Flavivirus* members than ELISA (Petersen et al., 2016; Staples et al., 2016). PRNT is a labor-intensive technique requiring a week for interpretation of results. Hence, a reporter virus possessing the luciferase gene from *Renilla* was used in a neutralization assay, and it showed similar specificity as PRNT, but reduced the time of diagnosis to 2 days (Shan et al., 2017a).

#### Reporter virus neutralization test (RVNT)

RVNT uses the same principle as that of PRNT but the difference in this test is the use of luciferase tagged ZIKV thus allowing the quantification of neutralization within 24 h instead of 7 days in PRNT. Similarly, this assay can be done on 96 well plates hence this assay minimizes the testing time and is a high throughput assay (Shan et al., 2017b).

#### Detection of specific IgG and IgM antibodies

Anti-ZIKV IgM can be detected after 5 days of onset of clinical symptoms and can last up to 3 months (Calvet et al., 2016). A recombinant ZIKV non-structural protein 1 (NS1) based-ELISA has been developed as an additional serological diagnostic which shows very low cross-reactivity with DENV antibodies, along with high specificity for ZIKV, and can be applied for serum-based diagnosis in pregnant women, travelers of ZIKA-endemic regions and individuals suspected of infection for counseling. The sensitivity was 58.8, 88.2, and 100% for IgM, IgG, and IgM/IgG respectively with 99.8% specificity (Steinhagen et al., 2016). Detection of specific IgG antibodies in maternal serum by ELISA represents an important approach to diagnose ZIKV infection and helps in determining the mechanism of neonatal microcephaly in congenital infection, even in mothers where ZIKV RNA has cleared (Sumita et al., 2016). A study using this ELISA was done on 105 serum samples collected from travelers returning to Israel. The sensitivity of the IgM ELISA differed among patients of Israeli origin and from European origin; hence, to minimize false negatives, further diagnostic tests are needed (Lustig et al., 2017). ZIKV-specific epitope-based serological assays can prevent the problem of cross-reactivity with other flavivirus antibodies (Landry and St. George, 2017). The IgM ELISA is a U.S. Food and Drug Administration (USFDA) approved assay for use with serum and CSF (Murray et al., 2017). A comparison of the commercially available NS1-based ELISA and the IgM ELISA showed better specificity with the IgM ELISA, but sensitivity was suboptimal during the first 5 days of infection (Kadkhoda et al., 2017). The competitive ELISA developed against NS1 of ZIKV, where out of confirmed RT-PCR positive 158 sera/plasma samples, 91.8% samples yielded higher than 50% inhibition (Balmaseda et al., 2017). The assay is robust, low cost strategy to surveillance program and seroprevalence studies and quickly adapted in ZIKV endemic areas.

#### Zika IgM antibody capture enzyme-linked immunosorbent assay (Zika MAC-ELISA)

The FDA has approved the use of CDC Zika IgM Antibody Capture Enzyme-Linked Immunosorbent Assay (Zika MAC-ELISA) for antibody testing in case of emergency. The technique is quantitative detection of ZIKV IgM antibodies in serum or CSF collected from patients. Result interpretation in MAC ELISA is based on the positive/Negative ratio (P/N) and the samples greater than or equal to 3 are considered to be “presumptive Zika IgM positive.” These samples are sent to approved laboratory for further confirmation by PRNT (Cordeiro, 2017; Landry and St. George, 2017). False positive results may be ruled out by checking with other methods like PRNT against Zika, dengue, and other flaviviruses (FDA, 2016).

#### Multiplex microsphere immunoassay (MIA)

The MIA detects antibodies against ZIKV surface protein E, non-structural NS1 and NS5 proteins in order to increase the accuracy and speed. It possesses improved serological diagnostic capability for ZIKV. MIA allows incorporation of more than one antigen to enhance the diagnostic coverage. The diagnostic width of MIA may further be improved by adding multiple antigens from ZIKV, DENV, YFV and other flaviviruses circulating in the same geographical area (Wong et al., 2017). A MIA has been developed for detection of 6 flaviviruses, 6 alphaviruses, and 1 bunyavirus of human importance (Basile et al., 2013). In this multiplex assay, ZIKV antigens may also be incorporated for developing ZIKV diagnostics.

#### Miscellaneous

A recently developed experimental murine model and ZIKV infection model in cynomolgus macaques may help to elucidate disease course, as well as would be useful in testing the anti-ZIKV drugs and vaccines (Koide et al., 2016; Rossi et al., 2016). In addition, a 3-[4,5-dimethyl-2-thiazolyl]-2,5-diphenyl-2H-tetrazolium bromide-(MTT)-based cell viability assay was developed to assess cell death in human and monkey cells caused by ZIKV. This has been claimed to be economic, simple, and rapid colorimetric assay that could be used for quantification of neutralizing antibodies against ZIKV (Müller et al., 2017).

The U.S. Centers for Disease Control and Prevention (CDC) Zika MAC-ELISA, InBios ZIKV *Detect*™MAC-ELISA, and Euroimmun anti-ZIKV IgM ELISA were compared in a study, and the CDC and InBios MAC-ELISAs were found to exhibit comparable sensitivities, whereas the Euroimmun ZIKV IgM ELISA was less sensitive (Granger et al., 2017). Recently, Euroimmun IgG/IgM ELISA was compared with PRNT and MAC-ELISA where it was found that Euroimmun ELISA had 92.5% specificity and 39.5% sensitivity when compared with MAC ELISA, while specificity ranged from 65 to 81% and sensitivity 83–92% when compared with PRNT (L'Huillier et al., 2017a); whereas Pasquier et al. (2018) revealed Diapro ZIKV IgG or IgM ELISA immunoassays more sensitive than the Euroimmun immunoassays.

Researchers have reported that MIA is more sensitive than IgM-capture ELISA for serological diagnosis of ZIKV infection. Moreover, the easy preparation, smaller sample volume requirement, and short time to get results are significant advantages of the MIA. Therefore, the proposed MIA is a useful tool for assessing immune responses in vaccination trials and in clinical disease (Wong et al., 2017). Recently, a multiplex diagnostic assay to detect DENV and ZIKV IgG, IgA, and IgM antibodies on a plasmonic gold platform have been developed (Zhang et al., 2017). An IgG/IgM based LFA, the DPP Zika IgM/IgG assay, marketed by Chembio Diagnostic Systems, is also available in the market (Nicolini et al., 2017). Electro-generated chemiluminescence was linked with polystyrene beads, which were also conjugated with monoclonal antibodies (mAbs) against ZIKV. This conjugated structure was used to capture ZIKV from fluids and detect up to one plaque-forming unit (PFU) of ZIKV in 100 μL of fluid (Acharya et al., 2016). A pair of mAbs has been selected to construct a rapid diagnostic test in form of strip. The diagnostic strip was able to detect NS1 antigen is serum samples from various geographic areas in the Americas and India. The same test is linked with mobile phone camera and the images taken can be analyzed with ImageJ software, which allow objective analysis and eliminates user subjectivity in reading test results (Bosch et al., 2017).

### Molecular diagnosis

#### RT-PCR

The ZIKV RNA has been reported in various body fluids such as blood, saliva, urine, and amniotic fluid (Hills et al., 2016; Rather et al., 2017) and hence it can be detected by RT-PCR. In a comparison of RT-PCR testing for ZIKV RNA in urine, serum, and saliva specimens obtained from people traveling in Florida and Brazil, urine samples were found to be the most suitable specimen for identifying acute infections with ZIKV (Bingham et al., 2016; Lamb et al., 2016). The RealStar Zika RT-PCR Test Kit, marketed by Altona Diagnostics, was found to be 91% sensitive and 97% specific in detecting ZIKV RNA; hence, this assay kit was proposed to diagnose early-stage ZIKV infection (L'Huillier et al., 2017b).

In a study, which tested a real-time RT-PCR based on the ZIKV envelope and NS2B genes was on urine and serum specimens from pregnant women, newborns, suspected symptomatic GBS patients, and travelers to ZIKV epidemic areas in New York. Due to very short viremia time period during ZIKV disease, there exists a challenge in selecting the appropriate sample for diagnosis, as ZIKV RNA may not be detected in serum or plasma collected 10 days after the onset of disease. Urine samples were found to be more reliable than serum samples, as the viral load was higher, and results of this assay were more dependable than those of serology (St. George et al., 2017). A study in Italy examined the whole blood, plasma and urine as samples for detection of RNA from 3rd day to 28 days post-ZIKV infection in 10 patients. Results showed that urine samples from all the individuals were ZIKV positive up to 21st day post-clinical sign while ZIKV could be detected in the whole blood up to 26 days (Rossini et al., 2017). During early disease, viral genomes could be detected in serum (Haug et al., 2016). ZIKV RNA has been detected in the breast milk of women, but there has been no report of transmission of ZIKV through breast milk. Another report showed that ZIKV persists for 81 days in whole blood, but 73 days in serum. Hence, whole blood can be used for ZIKV RNA detection in asymptomatic patients (Eppes et al., 2017; Murray et al., 2017). A dual target RT-PCR assay run on an automated m2000 system was developed to extract RNA from whole blood, serum, plasma, and urine and detect ZIKV by PCR. The detection limit of this assay was shown to be 120 copies/mL with whole blood, 30 copies/mL with serum, and 40 copies/mL with plasma and urine samples (Frankel et al., 2017).

Symptomatic pregnant women and affected persons display low-grade fever, maculopapular rashes, arthralgia, and non-purulent conjunctivitis for 2–7 days. Recently, in 2016–2017, asymptomatic pregnant women in American Samoa were tested under a syndromic surveillance program, with results displayed in electronic health records (Hancock et al., 2017).

A sensitive and specific one-step RT-PCR assay which includes GAPDH as internal control, which allows detection of PCR inhibitors in the sample, and rule out the possibility of false negatives was developed by Balm et al. (2012). The lower limit of detection of this assay was 140 RNA copies/PCR, with no cross-reactivity with closely related flaviviruses including DENV, YFV, and JEV.

A study was conducted using urine spiked with a commercially available heat-inactivated ZIKV panel (Exact Diagnostics® ZIKV Verification Panel), and several parameters were investigated, including the stability of ZIKV at different storage temperatures and the use of stabilizers for ZIKV RNA detection. The results showed that RNA degraded when stored at room temperature, whereas stability was better at 4°C. Storage of urine samples at −80°C led to decrease in RNA concentration. Similarly, the addition of nucleic acid stabilizers increased RNA recovery (Tan et al., 2017).

#### Real-time RT-PCR

Real time PCR offers several advantages over conventional RT-PCR including rapidity, low false positive, higher sensitivity and specificity with quantitative analysis. Blood and urine samples were collected and tested by real-time RT-PCR to confirm ZIKV infection (Hancock et al., 2017). Real-time RT-PCR can detect ZIKV RNA at an early stage in infection (Faye et al., 2008, 2013). ZIKV RNA can easily be detected in urine samples using real-time RT-PCR, with the added advantage of easy, non-invasive sample collection. This is especially useful during an epidemic and for the testing of travelers (Gourinat et al., 2015). Based on real-time RT-PCR analyses, several researchers have documented that ZIKV loads are higher in plasma than in urine samples (Pessôa et al., 2016). The probes developed against NS5 region in a real-time PCR and the sensitivity was 32 genome-equivalents and 0.05 plaque forming unit (pfu). The assay was able to detect at least 37 ZIKV isolates representatives of wide geographic area in Africa and Asia over the period of last 36 years. Also, it could differentiate ZIKV among 31 other flaviviruses. A SYBR Green one-step real-time RT-PCR assay detected ZIKV RNA, even at a titer of 1 PFU/ml; therefore, it is very useful for diagnosis and surveillance of ZIKV infection (Xu et al., 2016). Another study compared nine real-time RT-PCR assays and found that some assays were not suitable for the specific detection of ZIKV (Corman et al., 2016). A quantitative RT-PCR targeting E gene has been very recently developed that could detect all Asian and African lineages of ZIKV. This assay alleviates the disadvantage of earlier developed real-time PCR as they could not detect all strains of ZIKV and, moreover, this assay also showed a detection limit of 5 RNA transcript copies (Yang et al., 2017). A list of ZIKV detection assays is presented in Table 1.

**Table 1:** List of commercially available methodologies/kits for ZIKV diagnosis.

**S. No**.	**Diagnostic test/Technique**	**Sample(s)**	**Remarks**	**Demerits**	**References**
1	Electron microscopy	Human hepatic cells and neural progenitor cells,	- Drastic change in microtubules and intermediate filaments organization forming cage like structure around RF	- High purchase and maintenance cost - Highly stable voltage requirement - Sample preparation is time-consuming, labor-intensive and skill is required	Cortese et al., 2017
2	Neuroimaging	CT scan and MRI of prenatal fetus	- Polymicrogyria and lissencephaly-pachygyria - White-matter hypomyelination or dysmyelination with cerebellar hypoplasia - Calcified skull	- Expensive to use - In CT scan X-rays pass through different areas of brain - In MRI, the patient need to be still during imaging - MRI is incompatible with metallic devices like pacemakers	Zare Mehrjardi et al., 2016, 2017 Araujo Júnior et al., 2017 de Oliveira-Szejnfeld et al., 2017
3	Isolation of virus	Urine, saliva	- Cell lines like Vero cells, rhesus monkey kidney cells (LLC- 192 MK2), and mosquito origin [*A. albopictus* (C6/36), *A. psuedoscutellaris* (MOS61 or AP-61)] and lab animals can be used	- Needs specialized lab. - Takes several days for isolation	Bonaldo et al., 2016; Nikolay et al., in press
		Neural cells	- A129 type I interferon receptor knockout mice and AG129 type I and type II interferon receptor knockout mice	- Time consuming - Needs specialized facility to culture	Brault and Bowen, 2016
		Neuronal (SF268), retinal (ARPE19), pulmonary (Hep-2 and HFL), hepatic (Huh-7), placental (JEG-3), muscle (RD), and colonic (Caco-2) cell lines	- ZIKV replicate successfully	- Time consuming - Needs specialized facility to culture	Chan J. F.-W et al., 2016
4	IgM ELISA	Serum, CSF	- Zika MAC-ELISA- first FDA approved ZIKV diagnostic assay	- Cross reaction with other flavivirus. - Chance of false negative due to delayed seroconversion - Can be performed after 4 days of clinical signs	Staples et al., 2016
			- Highly specific	- Less sensitive during initial 5 days	Kadkhoda et al., 2017
5	Competitive ELISA for NS1	Serum, CSF	- Robust, low cost	- Sensitivity is high but specificity is low	Balmaseda et al., 2017
6	IgG ELISA	Serum	- IgG antibodies develop after IgM warning and stays life long	- Commercial kits not presently available	Eppes et al., 2017
			- NS1 based-ELISA highly sensitive for ZIKV and no cross reactivity with DENV	- Sensitivity only 58.8%,	Steinhagen et al., 2016
7	Plaque reduction neutralization test	Serum	- Less cross reactivity than ELISA	- Labor intensive - Requires a week time for result interpretation	Shan et al., 2017a
8	Multiplex Microsphere Immunoassay	Serum	- More sensitive that IgM ELISA - Small sample volume required and rapid completion rate	- Requires specialized equipment and correct antibody pair	Wong et al., 2017
9	IgM/IgG Lateral flow assay	Serum	- No cross-reactivity with other arboviruses	- Costly	Nicolini et al., 2017
10	Diagnostic strip test	Serum, urine, CSF	- Result analysis through ImageJ software installed inmobile phone camera - Allow objective analysis	- False positives may be obtained due to color of test sample	Bosch et al., 2017
11	RT-PCR	Serum, Whole blood, urine Tissues, CSF, amniotic Fluid, breast milk	- Rapid detection of ZIKV	- Requires sophisticated instruments	L'Huillier et al., 2017b; Murray et al., 2017
12	Real-time RT-PCR	Serum, Whole blood, urine Tissues, CSF, amniotic Fluid, breast milk	- Improved specificity and sensitivity	- Requires skilled labor and instruments	Gourinat et al., 2015
13	Real-time PCR-based endpoint assessment	Serum	- 100% sensitivity	- Takes 72 h for result interpretation	Wilson et al., 2017
14	RT- LAMP	Serum, Whole blood, urine Tissues, CSF, amniotic Fluid, breast milk	- Eliminates the requirement of sophisticated instruments. - No post-amplification modification	- Product carries over contamination. Hence closed tube techniques are developed	Wang et al., 2016
15	Surface plasmon resonance	Serum, Whole blood, urine Tissues, CSF, amniotic Fluid, breast milk	- Stronger fluorescence signals - Rapid and ultra-sensitive detection	- Higher cost	Adegoke et al., 2017
16	Flow cytometry	whole blood samples	- Detect as low as 0.5 MOI infection	- Higher running cost and instrument	Lum et al., 2017; Pardy et al., 2017
17	Paper-Based Plasmonic Biosensor	Serum	- Better stability at room temperature	- Higher running cost and instrument	Jiang et al., 2017; Morrissey et al., 2017
18	Sequencing	Any sample including environmental sample	- Amplify as less as 50 copies of viral genome- Fast analysis of genome	- Lot of repetitive sequence is generated	Quick et al., 2017

Real-time RT-PCR is a quick, reliable, sensitive and specific method. A comparison of 7 published real-time RT–PCR assays to determine the analytical sensitivity revealed presence of up to 10 potential mismatches in oligos with the Asian lineage. The two new assays developed by Corman et al. (2016) had 0–4 oligo mismatches. The continuous evolution in the viral genome and mismatch between oligos and genome may lead to reduced sensitivity. The study of 174 ZIKV genomes, exhibited the presence of mutation in diagnostic regions, however these were present in lower frequencies (Metsky et al., 2017). Hence, the diagnostic assay should be carefully chosen and need to be frequently evaluated and updated.

The co-circulation of other arboviruses hinders clear differential diagnosis of ZIKV (Pinto et al., 2015). A novel, highly sensitive and specific real-time RT-PCR assay that did not show cross-reactivity with other flaviviruses was developed by targeting the conserved 5′-untranslated region (5′-UTR), envelope (E), non-structural protein 2A (NS2A), NS5, and 3′-UTR of the ZIKV genome for improved laboratory diagnosis. It can detect even 5–10 RNA copies/reaction. Compared to the ZIKV-E gene targeting real-time PCR, the ZIKV-5'-UTR assay showed higher sensitivity in detecting ZIKV in most human tissues and the highest sensitivity in samples obtained from the testis/epididymis and kidney. This RT-PCR assay was found highly specific with no cross-reactivity with chikungunya virus, DENV, YFV, JEV, WNV, and hepatitis C virus (Chan et al., 2017).

#### Real-time PCR based neutralization assay

It is an assay into which the serum neutralization test and Real-time PCR tests are combined. The neutralization endpoint is measured by real -time PCR instead of counting plaques. The test takes 72 h to complete. This test may be employed as confirmatory test for those serum samples that are positive in an IgM/IgG ELISA. The sensitivity of this Real-time PCR based neutralization assay is 100% for both ZIKV and DENV (Wilson et al., 2017).

#### Surface plasmon resonance

Surface plasmon resonance-based technology for the sensitive detection of ZIKV RNA has been developed very recently wherein results revealed that bimetallic nanoparticle quantum dot-mediated fluorescent signals were stronger than those of single metal nanoparticles for the detection of ZIKV RNA (Adegoke et al., 2017). The technique is a reliable detection platform for rapid and ultra-sensitive detection of ZIKV genome. However, the higher cost of the test reduces its utility in low and middle-income group countries. A list of commercially available kits for ZIKV diagnosis is shown in Table 2.

**Table 2:** List of commercially available kits for ZIKV diagnosis.

**S. No**.	**Name of the kit**	**Marketed by**	**Remark**
**MOLECULAR ASSAYS**			
1	RealStar® Zika virus RT–PCR kit 1.0	Altona Diagnostics GmbH, Hamburg, Germany	91% sensitive and 97% specific
2	Genesig® Zika virus Advanced kit	Primerdesign Ltd, Birmingham, United Kingdom of Great Britain and Northern Ireland	Claimed to be highly specific
3	MyBioSource Zika real-time RT–PCR kit	MyBioSource Inc., San Diego, United States of America	ZIKV detection in serum and plasma
4	Zika Virus—Single Check	Genekam Biotechnology AG, Duisburg, German	Single check for Zika virus with detection limit 6.53 genome equivalent
5	FTD Zika virus RT–PCR kit	FastTrack Diagnostics, Esch-sur-Alzette, Luxembourg	One tube multiplex for detection of Zika virus and internal control
6	TaqMan Zika Virus Kit (ZIK	ThermoFisher Scientific	Single plex assay format designed to detect viral RNA, prepared from urine or serum samples, for Zika virus with Asian lineage
7	TaqPath Zika Virus Kit (ZIKV)		
8	TaqMan Zika Virus Triplex Kit		DetectsZIKV, DENV, CHIKV
9	TaqPath Zika Virus Triplex Kit		
10	Zika Virus (ZIKV) Real Time RT-PCR Kit	Liferiver Bio-Tech (United States) Corp.	–
11	TrioplexrRT-PCR	CDC	Whole blood, cerebrospinal fluid (CSF), urine, and amniotic fluid specimens may be used as starting material
			Detect ZIKV, DENV and chikungunya virus
12	AccuPower® RT- PCR Diagnostic Kit	Bioneer Corporation (South Korea)	Detect ZIKV, DENV and chikungunya virus in serum plasma and urine
13	Abbott RealTime Zika assay	Abbott Molecular Inc., (U.S.)	Detect ZIKVin serum, EDTA plasma, whole blood (EDTA), and urine
14	Zika Virus RNA Qualitative Real-Time RT-PCR	Quest Diagnostics, Inc., (U.S.)	Detect ZIKVin serum
15	Zika Virus Real-time RT-PCR kit	Viracor-IBT Laboratories, Inc., (U.S.)	Detect ZIKVin serum, plasma, or urine specimen
16	VERSANT® Zika RNA 1.0 Assay (kPCR) Kit	Siemens Healthcare Diagnostics Inc., (U.S.)	Detect ZIKVin serum, plasma, or urine with workflow efficiency
17	xMAP® MultiFLEX™ Zika RNA kit	Luminex Corporation, (U.S.)	Ability to run 1-96 samples in a single run and can be used with serum, plasma, or urine
18	Sentosa® SA ZIKV RT-PCR Test	Vela Diagnostics U.S., Inc., (U.S.)	Configured for automated workflow and detect ZIKVinserum, EDTA plasma or urine
19	Zika Virus Detection by RT-PCR Test	ARUP Laboratories, (U.S.)	Blood or urine
20	Gene-RADAR® Zika Virus Test	Nanobiosym Diagnostics, Inc., (U.S.)	Real time assay for ZIKV detection in serum
21	Genesig® Easy Kit	Primerdesign™ Ltd., (UK)	Broad dynamic detection range and Positive copy number standard curve for quantification is provided
22	RT-RPA	SRG (Zaghloul and El-shahat, 2014; Abd El Wahed et al., 2017)	Quick, no initial heating step, simple primer designing
23	RT-SIBA	SRG (Eboigbodin et al., 2016)	No detectable fluorescence in absence of target genome
			Detect as low as 10 copies of RNA
24	RT-LAMP	SRG (Chotiwan et al., 2017) SRG (Yaren et al., 2017) SRG (Tian et al., 2016)	Differentiation between African and Asian lineages of ZIKV is possible
			Use of thermolabile uracil DNA glycosylase to prevent carryover contamination
			Detection upto 1 attamole
**IMMUNOASSAYS**			
25	Zika virus IgG and IgM detection kits	MyBioSourceInc San Diego, USA	Double-antigen sandwich ELISA
26	Zika IgG/IgM Antibody Rapid Test	BiocanDiagnostics Inc. Coquitlam, Canada	Rapid finger-prick assay using NS1 protein and envelope protein detecting IgM and IgG antibodies
27	EUROIMMUN Anti-Zika Virus ELISA	Euroimmun AG, Lübeck, Germany	Immunofluorescence assay and ELISA for IgM and IgG
28	CDC Zika IgM Antibody Capture Enzyme-Linked Immunosorbent Assay (Zika MAC-ELISA)	U.S. Centers for Disease Control and Prevention	First FDA approved ZIKV detection assay. Detect Zika virus IgM antibodies in human serum or cerebrospinal fluid
29	DPP Zika IgM/IgG assay	Chembio Diagnostic Systems	Lateral flow assay
**MISCELLANEOUS STRATEGIES**			
30	Aptima Zika Virus assay	Hologic, Marlborough, MA	Detect virus in serum and urine
			Rapid and high-throughput method
31	Laser scanning assay	BluSense Diagnostics	Blue laser scanning of finger prick blood
32	Paper-based point-of-care test	Scientific researcher group (SRG) (Bedin et al., 2017)	Microfluidic paper-based analytical device is used
			Lower detection limit
33	TMA	SRG (Ren et al., 2017)	Rapid kinetics and high amplitude amplification within 15–60 min
34	RT-LAMP+LFA	SRG (Lee et al., 2016)	Detect even a single copy of ZIKV RNA in 35 min
35	NASBA coupled with CRISPR	SRG (Pardee et al., 2016)	Able to differentiate different strains of ZIKV

#### Flow cytometry

Flow cytometry technique is also used for the detection of ZIKV NS3 antigen in the whole blood samples using polyclonal antibodies. It has been reported recently that most patients revealed a decrease in ZIKV antigen during later phases of the disease while some showed higher antigen level. Flow cytometric study also revealed that CD14+ monocytes are the targets of ZIKV during acute phase of infection (Lum et al., 2017). Immature dendritic cells present in human skin cells are permissive to ZIKV infection and 24 h post-infection express viral envelope when infected with 0.5 multiplicity of infection (MOI). The intracellular presence of the viral envelope protein is detected by using a broadly neutralizing Ab 4G2 by flow cytometry. Also, the transcription level of RIG-I, MDA5, IRF7 and TLR3 expression is upregulated as soon as 6 hpi which lasts for 48 h. The role of these genes as sensor of infection may also have a role in the detection of ZIKV (Hamel et al., 2015). In ZIKV-infected mice, CD4+ T cells which have encountered ZIKV antigens, exhibit a typical Th1 cytokine profile distinguished with the production of IFN-γ, TNF-α, and IL-2. In infected mice, a higher proportion of CD11a+CD49d+ CD4+ T cells express the Th1 transcription factor (T-bet) in comparison to mock-infected mice (Pardy et al., 2017).

#### DNA sequencing

DNA sequencing provides confirmation of ZIKV infection (Zanluca et al., 2015). Sequencing of the NS5, NS3, and E genes can indicate the relationship between ZIKV strains (Fonseca et al., 2014; Grard et al., 2014; Tognarelli et al., 2015). Viral genomes may be enriched by multiplex PCR from samples containing as low as 50 copies of viral genome. Oligos were designed using Primal Scheme software and sequencing was done with the Oxford Nanopore MinION sequencing device. Clinical sample itself may be the starting material and within 1–2 days consensus viral sequence is obtained to do studies regarding evolution and spread of the virus (Quick et al., 2017). Multiple sequencing of 110 ZIKV genomes isolated from clinical and mosquito samples from 10 countries and territories, was done and compared with other 64 published genomes from NCBI GenBank which revealed that ZIKV outbreak has started from Brazil (Metsky et al., 2017). Deep sequencing can be employed to analyze the ZIKV genome, and a comparison with sequences available in a data bank can help to identify nucleotide changes in specific strains of the virus (Buechler et al., 2017). More recently, by employing next generation sequencing the whole genome sequence of ZIKV strain AFMC-U has been amplified from the urine sample of a Korean traveler. This report also shows the suitability and importance of urine as a sample for ZIKV diagnosis (Gu et al., 2017).

### Other advanced diagnostics

#### Reverse transcription isothermal recombinase polymerase amplification

A reverse transcription (RT) isothermal recombinase polymerase amplification assay (RT-RPA) that targets the NS2A region with 92% sensitivity and 100% specificity compared to RT-PCR has been developed (Abd El Wahed et al., 2017). It is a rapid assay which takes only 3–15 min and able to detect 21 RNA molecules. RPA shows many advantages over both real-time PCR and other isothermal amplification methods including Nucleic acid sequence-based amplification (NASBA), LAMP, Strand-displacement amplification (SDA), Rolling circle amplification (RCA), and Helicase-dependent amplification (HAD) in power saving (runs at 37°C), simple primer designing, quickness, no initial heating step and robustness for biological substances (Zaghloul and El-shahat, 2014).

#### Transcription-mediated amplification (TMA) technology

The technology uses two enzymes, reverse transcriptase (RT) and RNA polymerase. It produces RNA amplicons, opposite to other assays which produce DNA and have rapid kinetics, which result in higher amplitude amplification within 15–60 min. The TMA technology-based Aptima Zika Virus assay (Hologic, Marlborough, MA) runs on automated Panther system and has shown 94.7% sensitivity and 94.8% specificity relative to that of real-time RT-PCR (Ren et al., 2017). A paper-based point-of-care test was developed very recently to detect multiple flaviviruses, and a microfluidic paper-based analytical device (μPAD) has been used to detect as little as 10 ng/ml of NS1 protein in plasma and blood. This assay is economic, specific, and provides a result in less than 8 min (Bedin et al., 2017).

#### Real-time loop-mediated isothermal amplification (RT-LAMP)

Researchers have developed a one-step RT-LAMP technique that targets the entire ZIKV genome and shows high sensitivity and specificity. The RT-LAMP assay was compared with conventional and quantitative real-time RT-PCR, and results revealed excellent sensitivity and specificity in the detection of ZIKV (Wang et al., 2016). A LAMP assay to distinguish between African and Asian lineages of ZIKV has been developed to detect ZIKV RNA from mosquitoes, virus-spiked samples, human saliva, blood, plasma, and urine without RNA extraction (Chotiwan et al., 2017). The LAMP assay has the disadvantage of product carry-over contamination; hence, closed-tube techniques are usually preferred (Karthik et al., 2014). Recently, a LAMP assay to detect ZIKV was developed that utilizes a dTTP-dUTP mix with a thermolabile uracil DNA glycosylase that can prevent carryover contamination (Yaren et al., 2017). An RT-LAMP without a microfluidic cassette for the detection of ZIKV has been introduced as a point-of-care diagnostic assay (Song et al., 2016). An RT-LAMP assay combined with an LFA was developed to detect even a single copy of ZIKV RNA, with highly specific results available within 35 min (Lee et al., 2016). In addition, a smartphone-based RT-LAMP assay for easy detection of ZIKV in human saliva, urine, and blood was created. This assay used a portable LAMP box powered by a 5 V power source and a smartphone built using a chromaticity algorithm to scan for fluorescent signals. Use of this technology increased the sensitivity of this assay 5-folds compared to that of naked eye detection of colorimetric results (Priye et al., 2017). Finally, a portable device was developed that can extract nucleic acids using magnetic particles, then perform real-time RT-RPA or RT-PCR and interpret results by fluorescence detection. A 3D printer has also been used to process 8–12 samples for the detection of ZIKV, with fluorescence detection of amplified products by smart phone. This assay is cheap and portable (Chan K. et al., 2016). A higher sensitivity was obtained by Tian et al. (2016); they achieved a detection limit of one attomole using a synthetic ZIKV oligonucleotide within 27 min in serum.

#### Nucleic acid sequence-based amplification (NASBA)

Recently, a novel diagnostic assay has been developed that employs biomolecular sensors to colorimetrically detect ZIKV RNA by nucleic acid sequence-based amplification (NASBA). It also uses CRISPR technology that can differentiate between ZIKV strains. The coupled NASBA and CRISPR detection module was able to discriminate viral strains differing by one base and also able to differentiate the African ZIKV strain with that of American strains. The programmability of molecular sensors will help in addressing rapidly changing diagnostic requirements (Pardee et al., 2016).

#### Reverse transcription strand invasion based amplification (RT-SIBA) assay

RT strand invasion-based amplification by a battery-operated portable device for the diagnosis of ZIKV was reported to work well, even with incompletely purified RNA. It relies on a recombinase coated single-stranded invasion oligonucleotide purposed to separate complementary target duplex. Resulting single-stranded target template is extended by DNA polymerase at a relative low and constant temperature. The method is simple and can run on low cost equipments. The advantage of RT-SIBA includes no production of detectable fluorescence signal if the target template is absent, where in real-time RT-PCR a detectable signal is produced after 30 threshold cycles (Ct). Also, it is able to reproducibly detect as low as 10 copies of RNA (Eboigbodin et al., 2016).

#### Bioplasmonic paper-based device (BPD)

The technique is based on the detection of IgG and IgM antibodies to NS1 protein of ZIKV. ZIKV-NS1 protein here acts as capturing element and gold nanorods act as plasmonic nano-transducers (Jiang et al., 2017). The technique offers and excellent stability of the BPD at room temperature and even at higher temperatures using metal–organic framework dependent biopreservation. The technique eliminates the storage and transportation of device at low temperature (Morrissey et al., 2017), thus having field applicability. The same device may be adapted for diagnosis of other infectious agents also.

#### Other technologies

Other recent technologies for ZIKV diagnosis include paper disk tools, which work on the principle of a color change from yellow to purple in the presence of ZIKV antigen (Shukla et al., 2016). Recent techniques like “toehold switches” that can bind to any sense RNA sequences can also be applied for ZIKV diagnosis (Pardee et al., 2016). To concentrate ZIKV in samples with low viral loads, ultracentrifugation and polyethylene glycol precipitation can be performed, but these methods have their own limitations and can interfere with PCR assays (Novotny et al., 1992). Therefore, recently, magnetic nanoparticle-based concentration has been preferred. Nanoparticles like such as iron, nickel, and cobalt can be used, but their stability is poor; hence, they are encapsulated with silica, graphite, or a polymer (Saraswati et al., 2012). This method has been used for DENV detection using a PCR assay that can also be employed to detect ZIKV (Sakudo et al., 2016). A liposome-based biosensor that is cheap, portable, specific, and sensitive has already been developed for DENV (Zaytseva et al., 2004). These platforms can also be utilized for the detection of ZIKV. Very recently surface-enhanced Raman spectroscopy (SERS) was utilized to detect ZIKV and DENV in a multiplexed assay platform. To improve the sensitivity of LFA, SERS has been combined so as to increase the ZIKV diagnosis along with differentiation from DENV (Sánchez-Purrà et al., 2017).

**Figure 2:**
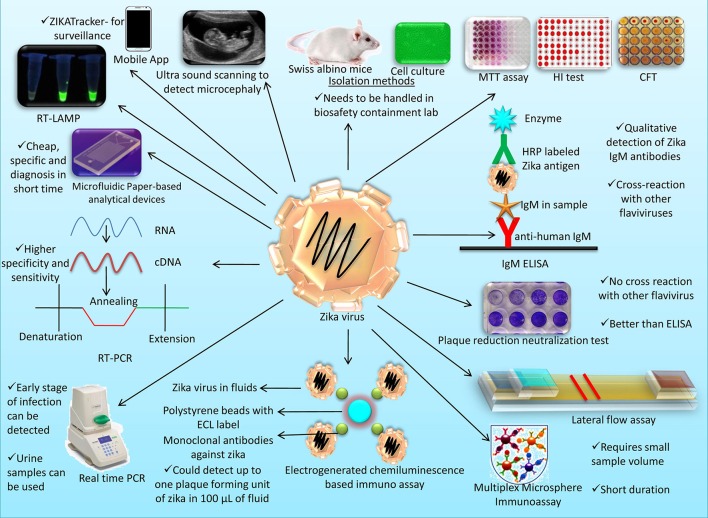
An overview on different diagnostic platforms available for Zika virus detection.

Different diagnostic platforms available for Zika virus detection are presented in Figure 2.

### ZIKV surveillance strategies

Extensive ZIKV epidemiological studies should be carried out to support advanced disease surveillance and monitoring approaches, and geographical information system (GIS) and appropriate networking programs should be employed to track the spread of the virus (Dhama et al., 2013; Shukla et al., 2016; Sharma and Lal, 2017). Based on the surveillance data of the Zika epidemics in the Tolima department, Colombia, epidemiological mapping has been developed employing GIS. Kosmo Desktop 3.0RC1® was used as the GIS software in this study and it was found that eastern area of this department was highly affected with ZIKV (Rodriguez-Morales et al., 2016). Similarly other reports also show that GIS was employed to develop epidemiological mapping in Valle del Cauca and Pereira department of Colombia. It was found that North part of Valle del Cauca and South-West part of Pereira department had most of the ZIKV burden (Rodriguez-Morales et al., 2017a,b). A mobile App (ZIKATracker, zikatracker.net) has recently been developed to report the occurrence of cases, which can be helpful for effective and timely control of the disease (Kelvin et al., 2016). Early reporting of ZIKV cases can allow the pattern of disease spread to be determined, as well as limit its further spread (Nishiura et al., 2016). Efficient serological and molecular detection techniques are warranted for improved surveillance to prevent the spread of disease by adopting timely and appropriate control measures (Waggoner and Pinsky, 2016; Sharma and Lal, 2017). In North America, the GeoSentinel Surveillance Network data platform has been used to track Canadian travelers displaying signs of acute ZIKV infection and fetal neurologic anomalies between October 2015 and September 2016 on the CanTravNet site (Boggild et al., 2017).

When the recent ZIKV outbreak occurred in South and Central American states, several national and international organizations expressed their concern for the safety of athletes, players, coaches, and viewers during the Rio Olympic and Paralympic games. Even blood and urine samples from asymptomatic persons were tested by real-time RT-PCR (Shadgan et al., 2016). Epidemiological studies confirmed that, after 2015, approximately 1.5 million people in Brazil alone were infected with ZIKV, with almost 80% of cases asymptomatic but positive for viral RNA. Since 2007, more than 55 countries in the Americas, Asia, Africa, the Caribbean, and the Pacific region have reported cases with GBS syndrome, retinal/eye lesions, and/or increased incidence of microcephaly that were confirmed by RT-PCR, IgM ELISA, or neutralizing antibody-based serological tests (Carod-Artal, 2016; Pyzocha et al., 2017).

Past vaccination against another flavivirus or a recent infection with a flavivirus may interfere with serological testing, increasing the probability of false positive results. In addition, these tests are time-consuming, laborious, and cross-reactivity with antibodies against other flaviviruses may lead to a misinterpretation of results. Hence, molecular tests are preferred over serological approaches. A number of nucleic acid amplification-based tests have been used to detect even acute infections by specifically replicating ZIKV RNA. However, in the case of asymptomatic patients or convalescent-phase samples, nucleic acid based techniques have limitations; therefore, specific and sensitive antibody-based tests that can detect ZIKV-specific epitopes or anti-ZIKV antibody are needed for diagnosis (Landry and St. George, 2017).

Advanced detection techniques include multiplex PCR, LAMP, recombinant diagnostics, biosensors/biochips, microarrays, and nanodiagnostics (Ratcliff et al., 2007; Bergquist, 2011; Kawadkar et al., 2011; Dhama et al., 2014; Van den Hurk and Evoy, 2015; Lambe et al., 2016). WHO declared the Zika epidemic a public health emergency of international concern, which emphasized need for rapid, accurate, cost-effective, and sensitive point-of-care diagnostics to prevent infection and its health effects of GBS and microencephaly in developing fetuses (Calvet et al., 2016; Shukla et al., 2016). Along with developing rapid and confirmatory diagnostics, discovering drugs, medicines, therapeutics, and vaccines, as well as effective prevention and control interventions, is necessary to safeguard the health of humans from this important pathogen of global importance (Koff et al., 2013; Singh et al., 2015; Khandia et al., 2017; Munjal et al., 2017a,b).

## Conclusions and future perspectives

ZIKV existence is known since 1947, but, until recently, it has only been associated with mild disease. Nonetheless, its recent emergence has presented a pandemic threat world over with the appearance of increased cases of neurological anomalies like GBS and microcephaly. Hence, researchers across the world emphasized targeted to develop rapid confirmatory diagnostics and efficient drugs and medicines, as well as design appropriate prevention and control strategies to curtail ZIKV spread and associated ill effects. Understanding the factors responsible for the apparent greater pathogenicity of ZIKV infection by in-depth molecular exploration on the virus and host-pathogen interactions would help in designing effective diagnostics, drugs, and control strategies to counter ZIKV. Pre-existing antibodies to DENV may cross-react with ZIKV and, through the phenomenon of antibody-dependent enhancement, and result in an increase in the virus titer and enhanced ZIKV infection. Exploiting recent knowledge gained in immunology, biotechnology, and molecular biology, there is need to develop accurate and rapid detection assays for ZIKV/Zika fever, so as detailed disease informatics could be ascertained with an early and timely implementation of disease prevention and control measures. A better understanding of ZIKV pathogenesis and genetics would allow novel targets to be identified for the design and development of effective and safer drugs, medicines, pharmaceuticals, and vaccines to counter ZIKV effectively. Stronger and more extensive ZIKV surveillance, monitoring, and networking programs need to be implemented to prevent additional ZIKV emergencies in the future.

## Author contributions

All the authors substantially contributed to the conception, design, analysis and interpretation of data, checking and approving final version of manuscript, and agree to be accountable for its contents. RS designed an overview on virological and molecular diagnosis; KD, RT, KK, RK, and AM updated different aspects regarding serological and recent diagnostic advances; KK and RK designed tables and KK designed the figures; RS, KD, YM, HI, and RB-M finally reviewed, analyzed, edited and concluded the review compilation.

### Conflict of interest statement

The authors declare that the research was conducted in the absence of any commercial or financial relationships that could be construed as a potential conflict of interest.

## References

[B1] Abd El Wahed A. Sanabani S. S. Faye O. Pessôa R. Patriota J. V. Giorgi R. R.. (2017). Rapid molecular detection of Zika virus in acute-phase urine samples using the recombinase polymerase amplification assay. PLoS Curr. 9:ecurrents.outbreaks.a7f1db2c7d66c3fc0ea0a774305d319e. 10.1371/currents.outbreaks.a7f1db2c7d66c3fc0ea0a774305d319e 28239513 PMC5309125

[B2] Acharya D. Bastola P. Le L. Paul A. M. Fernandez E. Diamond M. S.. (2016). An ultrasensitive electrogenerated chemiluminescence-based immunoassay for specific detection of Zika virus. Sci. Rep. 6:32227. 10.1038/srep32227 27554037 PMC4995374

[B3] Adegoke O. Morita M. Kato T. Ito M. Suzuki T. Park E. Y. (2017). Localized surface plasmon resonance-mediated fluorescence signals in plasmonic nanoparticle-quantum dot hybrids for ultrasensitive Zika virus RNA detection via hairpin hybridization assays. Biosens. Bioelectron. 94, 513–522. 10.1016/j.bios.2017.03.046 28343104

[B4] Al-Qahtani A. A. Nazir N. Al-Anazi M. R. Rubino S. Al-Ahdal M. N. (2016). Zika virus: a new pandemic threat. J. Infect. Dev. Ctries. 10, 201–207. 10.3855/jidc.8350 27031450

[B5] Araujo Júnior E. Carvalho F. H. Tonni G. Werner H. (2017). Prenatal imaging findings in fetal Zika virus infection. Curr. Opin. Obstet. Gynecol. 29, 95–105. 10.1097/GCO.0000000000000345 28134669

[B6] Balm M. N. D. Lee C. K. Lee H. K. Chiu L. Koay E. S. C. Tang J. W. (2012). A diagnostic polymerase chain reaction assay for Zika virus. J. Med. Virol. 84, 1501–1505. 10.1002/jmv.23241 22825831

[B7] Balmaseda A. Stettler K. Medialdea-Carrera R. Collado D. Jin X. Zambrana J. V.. (2017). Antibody-based assay discriminates Zika virus infection from other flaviviruses. Proc. Natl. Acad. Sci U.S.A. 114, 8384–8389. 10.1073/pnas.1704984114 28716913 PMC5547631

[B8] Barzon L. Pacenti M. Berto A. Sinigaglia A. Franchin E. Lavezzo E.. (2016). Isolation of infectious Zika virus from saliva and prolonged viral RNA shedding in a traveller returning from the Dominican Republic to Italy, January 2016. Euro Surveill. 21, 1–5. 10.2807/1560-7917.ES.2016.21.10.30159 26987769

[B9] Basile A. J. Horiuchi K. Panella A. J. Laven J. Kosoy O. Lanciotti R. S.. (2013). Multiplex microsphere immunoassays for the detection of IgM and IgG to arboviral diseases. PLoS ONE 8:e75670. 10.1371/journal.pone.0075670 24086608 PMC3783417

[B10] Bedin F. Boulet L. Voilin E. Theillet G. Rubens A. Rozand C. (2017). Paper-based point-of-care testing for cost-effective diagnosis of acute flavivirus infections. J. Med. Virol. 89, 1520–1527. 10.1002/jmv.24806 28295400

[B11] Bergquist R. (2011). New tools for epidemiology: a space odyssey. Mem. Inst. Oswaldo Cruz. 106, 892–900. 10.1590/S0074-02762011000700016 22124563

[B12] Bingham A. M. Cone M. Mock V. Heberlein-Larson L. Stanek D. Blackmore C.. (2016). Comparison of test results for Zika virus RNA in Urine, Serum, and Saliva specimens from persons with travel-associated Zika virus disease - Florida, 2016. MMWR Morb. Mortal. Wkly. Rep. 65, 475–478. 10.15585/mmwr.mm6518e2 27171533

[B13] Boggild A. K. Geduld J. Libman M. Yansouni C. P. McCarthy A. E. Hajek J.. (2017). Surveillance report of Zika virus among Canadian travellers returning from the Americas. CMAJ. 189, E334–E340. 10.1503/cmaj.161241 28280063 PMC5334005

[B14] Bonaldo M. C. Ribeiro I. P. Lima N. S. Dos Santos A. A. Menezes L. S. da Cruz S. O.. (2016). Isolation of infective Zika virus from urine and Saliva of patients in Brazil. PLoS Negl. Trop. Dis. 10:e0004816. 10.1371/journal.pntd.0004816 27341420 PMC4920388

[B15] Bosch I. de Puig H. Hiley M. Carré-Camps M. Perdomo-Celis F. Narváez C. F.. (2017). Rapid antigen tests for dengue virus serotypes and Zika virus in patient serum. Sci. Transl. Med. 9:eaan1589. 10.1126/scitranslmed.aan1589 28954927 PMC6612058

[B16] Brault A. C. Bowen R. A. (2016). The development of small animal models for Zika virus vaccine efficacy testing and pathological assessment. Am. J. Trop. Med. Hyg. 94, 1187–1188. 10.4269/ajtmh.16-0277 27139439 PMC4889731

[B17] Buechler C. R. Bailey A. L. Weiler A. M. Barry G. L. Breitbach M. E. Stewart L. M.. (2017). Seroprevalence of Zika virus in wild African green monkeys and baboons. mSphere. 2:e00392-16. 10.1128/mSphere.00392-16 28289727 PMC5343173

[B18] Calvet G. A. Santos F. B. Sequeira P. C. (2016). Zika virus infection: epidemiology, clinical manifestations and diagnosis. Curr. Opin. Infect. Dis. 29, 459–466. 10.1097/QCO.0000000000000301 27496713

[B19] Cao-Lormeau V. M. Blake A. Mons S. Lastère S. Roche C. Vanhomwegen J.. (2016). Guillain-Barré Syndrome outbreak associated with Zika virus infection in French Polynesia: a case-control study. The Lancet. 387, 1531–1539. 10.1016/S0140-6736(16)00562-6 26948433 PMC5444521

[B20] Carod-Artal F. J. (2016). Epidemiology and neurological complications of infection by the Zika virus: a new emerging neurotropic virus. Rev. Neurol. 62, 317–328. 26988170

[B21] Carteaux G. Maquart M. Bedet A. Contou D. Brugières P. Fourati S.. (2016). Zika virus associated with meningoencephalitis. N. Engl. J. Med. 374, 1595–1596. 10.1056/NEJMc1602964 26958738

[B22] Chan J. F. Yip C. C. Tee K. M. Zhu Z. Tsang J. O. Chik K. K.. (2017). Improved detection of Zika virus RNA in human and animal specimens by a novel, highly sensitive and specific real-time RT-PCR assay targeting the 5'-untranslated region of Zika virus. Trop. Med. Int. Health. 22, 594–603. 10.1111/tmi.12857 28214373

[B23] Chan J. F.-W. Yip C. C. Tsang J. O. Tee K. M. Cai J. P. Chik K. K.. (2016). Differential cell line susceptibility to the emerging Zika virus: implications for disease pathogenesis, non-vector-borne human transmission and animal reservoirs. Emerg. Microbes Infect. 5:e93. 10.1038/emi.2016.99. 27553173 PMC5034105

[B24] Chan K. Weaver S. C. Wong P. Y. Lie S. Wang E. Guerbois M.. (2016). Rapid, affordable and portable medium-throughput molecular device for Zika virus. Sci. Rep. 6:38223. 10.1038/srep38223 27934884 PMC5146750

[B25] Chotiwan N. Brewster C. D. Magalhaes T. Weger-Lucarelli J. Duggal N. K. Rückert C.. (2017). Rapid and specific detection of Asian- and African-lineage Zika viruses. Sci. Transl. Med. 9:eaag0538. 10.1126/scitranslmed.aag0538 28469032 PMC5654541

[B26] Coelho S. V. A. Neris R. L. S. Papa M. P. Schnellrath L. C. Meuren L. M. Tschoeke D. A.. (2017). Development of standard methods for Zika virus propagation, titration, and purification. J. Virol. Methods. 246, 65–74. 10.1016/j.jviromet.2017.04.011 28445704

[B27] Cordeiro M. T. (2017). Zika virus: laboratory diagnosis, in Zika in Focus, ed Vasco Aragão M. (Cham: Springer), 9–62.

[B28] Corman V. M. Rasche A. Baronti C. Aldabbagh S. Cadar D. Reusken C. B. E. M.. (2016). Assay optimization for molecular detection of Zika virus. Bull. World Health Organ. 94, 880–892. 10.2471/BLT.16.175950 27994281 PMC5153932

[B29] Cortese M. Goellner S. Acosta E. G. Neufeldt C. J. Oleksiuk O. Lampe M.. (2017). Ultrastructural characterization of Zika virus replication factories. Cell Rep. 18, 2113–2123. 10.1016/j.celrep.2017.02.014 28249158 PMC5340982

[B30] de Fatima Vasco Aragao M. van der Linden V. Brainer-Lima A. M. Coeli R. R. Rocha M. A. Sobral da Silva P.. (2016). Clinical features and neuroimaging (CT and MRI) findings in presumed Zika virus related congenital infection and microcephaly: retrospective case series study. BMJ. 353, i1901. 10.1136/bmj.i3182 27075009 PMC4830901

[B31] de M. Campos R. Cirne-Santos C. Meira G. L. S. Santos L. L. R. de Meneses M. D. Friedrich J. (2016). Prolonged detection of Zika virus RNA in urine samples during the ongoing Zika virus epidemic in Brazil. J. Clin. Virol. 77, 69–70. 10.1016/j.jcv.2016.02.009 26921737

[B32] de Oliveira-Szejnfeld P. S. Levine D. Melo A. S. D. O. Amorim M. M. R. Batista A. G. M. Chimelli L. (2017). Congenital brain abnormalities and Zika virus: what the radiologist can expect to see prenatally and postnatally. Radiology 281, 203–218. 10.1148/radiol.2016161584 27552432

[B33] Dhama K. Karthik K. Chakraborty S. Tiwari R. Kapoor S. Kumar A.. (2014). Loop-mediated isothermal amplification of DNA (LAMP) – a new diagnostic tool lights the world of diagnosis of animal and human pathogens: a review. Pak. J. Biol. Sci. 17, 151–166. 10.3923/pjbs.2014.151.166 24783797

[B34] Dhama K. Verma A. K. Tiwari R. Chakraborty S. Vora K. Kapoor S. (2013). A perspective on applications of geographical information system (GIS); an advanced tracking tool for disease surveillance and monitoring in veterinary epidemiology. Adv. Anim. Vet. Sci. 1, 14–24.

[B35] Eboigbodin K. E. Brummer M. Ojalehto T. Hoser M. (2016). Rapid molecular diagnostic test for Zika virus with low demands on sample preparation and instrumentation. Diag. Microb. Infect. Dis. 86, 369–371. 10.1016/j.diagmicrobio.2016.08.027 27645608

[B36] Eppes C. Rac M. Dunn J. Versalovic J. Murray K. O. Suter M. A.. (2017). Testing for Zika virus infection in pregnancy: key concepts to deal with an emerging epidemic. Am. J. Obstet. Gynecol. 216, 209–225. 10.1016/j.ajog.2017.01.020 28126366

[B37] Fagbami A. H. (1979). Zika virus infections in Nigeria: virological and seroepidemiological investigations in Oyo State. J. Hyg. 83, 213–219. 10.1017/S0022172400025997 489960 PMC2129900

[B38] Fajardo A. Cristina J. Moreno P. (2016). Emergence and spreading potential of Zika virus. Front. Microbiol. 7:1667. 10.3389/fmicb.2016.01667 27812357 PMC5071320

[B39] Faye O. Faye O. Diallo D. Diallo M. Weidmann M. Sall A. A. (2013). Quantitative real-time PCR detection of Zika virus and evaluation with field-caught mosquitoes. Virol. J. 10, 311–318. 10.1186/1743-422X-10-311 24148652 PMC4016539

[B40] Faye O. Faye O. Dupressoir A. Weidmann M. Ndiaye M. Alpha Sall A. (2008). One-step RT-PCR for detection of Zika virus. J. Clin. Virol. 43, 96–101. 10.1016/j.jcv.2008.05.005 18674965

[B41] FDA (2016). Zika Virus Emergency Use Authorization. Silver Spring, MD: US Department of Health and Human Services, Food and Drug Administration Available online at: http://www.fda.gov/MedicalDevices/Safety/EmergencySituations/ucm161496.htm

[B42] Fonseca K. Meatherall B. Zarra D. Drebot M. MacDonald J. Pabbaraju K.. (2014). First case of Zika virus infection in a returning Canadian traveler. Am. J. Trop. Med. Hyg. 91, 1035–1038. 10.4269/ajtmh.14-0151 25294619 PMC4228871

[B43] Frankel M. B. Pandya K. Gersch J. Siddiqui S. Schneider G. J. (2017). Development of the Abbott RealTime ZIKA assay for the qualitative detection of Zika virus RNA from serum, plasma, urine, and whole blood specimens using the m2000 system. J. Virol. Methods 246, 117–124. 10.1016/j.jviromet.2017.05.002 28479349

[B44] Fung I. C. Blankenship E. B. Goff M. E. Mullican L. A. Chan K. C. Saroha N. (2017). Zika-virus-related photo sharing on Pinterest and Instagram. Disaster Med. Public Health Prep. 23, 1–4. 10.1017/dmp.2017.23 28330514

[B45] Gourinat A. C. O'Connor O. Calvez E. Goarant C. Dupont-Rouzeyrol M. (2015). Detection of Zika virus in urine. Emerging Infect. Dis. 21, 84–86. 10.3201/eid2101.140894 25530324 PMC4285245

[B46] Granger D. Hilgart H. Misner L. Christensen J. Bistodeau S. Palm J. (2017). Serologic testing for zika virus: comparison of three Zika virus igm elisas and initial laboratory experiences. J. Clin. Microbiol. 55, 2127–2136. 10.1128/JCM.00580-17 28446573 PMC5483914

[B47] Grard G. Caron M. Mombo I. M. Nkoghe D. Mboui Ondo S. Jiolle D.. (2014). Zika virus in Gabon (Central Africa) 2007: a new threat from *Aedes albopictus*? PLoS Negl. Trop. Dis. 8:e2681. 10.1371/journal.pntd.0002681 24516683 PMC3916288

[B48] Gu S. H. Song D. H. Lee D. Jang J. Kim M. Y. Jung J.. (2017). Whole-genome sequence analysis of Zika virus, amplified from urine of traveler from the Philippines. Virus Genes 53, 918–921. 10.1007/s11262-017-1500-9 28795266 PMC5698360

[B49] Haddow A. J. Williams M. C. Woodall J. P. Simpson D. I. Goma L. K. (1964). Twelve isolations of Zika virus from Aedes (Stegomyia) africanus (theobald) taken in and above a Uganda forest. Bull. World Health Organ. 31, 57–69. 14230895 PMC2555143

[B50] Hamel R. Dejarnac O. Wichit S. Ekchariyawat P. Neyret A. Luplertlop N. (2015). Biology of Zika virus infection in human skin cell. J. Virol. 89, 8880–8896. 10.1128/JVI.00354-15 26085147 PMC4524089

[B51] Hamel R. Liégeois F. Wichit S. Pompon J. Diop F. Talignani L.. (2016). Zika virus: epidemiology, clinical features and host-virus interactions. Microbes Infect. 18, 441–449. 10.1016/j.micinf.2016.03.009 27012221

[B52] Hancock W. T. Soeters H. M. Hills S. L. Link-Gelles R. Evans M. E. Daley W. R.. (2017). Establishing a timeline to discontinue routine testing of asymptomatic pregnant women for Zika virus infection - American samoa, 2016-2017. MMWR Morb. Mortal. Wkly. Rep. 66, 299–301. 10.15585/mmwr.mm6611a5 28333910 PMC5657887

[B53] Haug C. J. Kieny M. P. Murgue B. (2016). The Zika challenge. N. Engl. J. Med. 374, 1801–1803. 10.1056/NEJMp1603734 27028782

[B54] Hayes E. B. (2009). Zika virus outside Africa. Emerg. Infect. Dis. 15, 1347–1350. 10.3201/eid1509.090442 19788800 PMC2819875

[B55] Hills S. L. Russell K. Hennessey M. Williams C. Oster A. M. Fischer M.. (2016). Transmission of Zika virus through sexual contact with travelers to areas of ongoing transmission–continental United States, 2016. MMWR Morb. Mortal. Wkly. Rep. 65, 215–216. 10.15585/mmwr.mm6508e2 26937739

[B56] Ioos S. Mallet H. P. Leparc Goffart I. Gauthier V. Cardoso T. Herida M. (2014). Current Zika virus epidemiology and recent epidemics. Med. Mal. Infect. 44, 302–307. 10.1016/j.medmal.2014.04.008 25001879

[B57] Jacob J. A. (2016). Researchers focus on solving the Zika riddles. JAMA. 315, 1097–1099. 10.1001/jama.2016.1219 26914596

[B58] Jiang Q. Chandar Y. J. Cao S. Kharasch E. D. Singamaneni S. Morrissey J. J. (2017). Rapid, point-of-care, paper-based plasmonic biosensor for Zika virus diagnosis. Adv. Biosys. 1:1700096 10.1002/adbi.201700096 32646188

[B59] Kadkhoda K. Gretchen A. Racano A. (2017). Evaluation of a commercially available Zika virus IgM ELISA: specificity in focus. Diagn. Microbiol. Infect. Dis. 88, 233–235. 10.1016/j.diagmicrobio.2017.04 28478111

[B60] Karthik K. Rathore R. Thomas P. Arun T. R. Viswas K. N. Dhama K.. (2014). New closed tube loop mediated isothermal amplification assay for prevention of product cross contamination. MethodsX 1, e137–e143. 10.1016/j.mex.2014.08.009 26150945 PMC4472950

[B61] Kawadkar J. Chauhan M. K. Maharana M. (2011). Nanobiotechnology: application of nanotechnology in diagnosis, drug discovery and drug development. Asian Pharmaceut Clin Res. 4, 23–28.

[B62] Kelvin A. A. Banner D,. Pamplona L. Alencar C. Rubino S. Heukelbach J. (2016). ZIKATracker: a mobile App for reporting cases of ZIKV worldwide. J. Infect. Dev. Ctries. 10, 113–115. 10.3855/jidc.8248 26927449

[B63] Khandia R. Munjal A. Dhama K. (2017). Consequences of Zika virus infection during fetal stage and pregnancy safe drugs: an update. Int. J. Pharmacol. 13, 370–377. 10.3923/ijp.2017.370.377

[B64] Kirya B. G. Okia N. O. (1977). A yellow fever epizootic in Zika Forest, Uganda, during 1972: Part 2: monkey serology. Trans. R. Soc. Trop. Med. Hyg. 71, 300–303. 10.1016/0035-9203(77)90104-3 413216

[B65] Koff W. C. Burton D. R. Johnson P. R. Walker B. D. King C. R. Nabel G. J.. (2013). Accelerating next generation vaccine development for global disease prevention. Science 340:1232910. 10.1126/science.1232910 23723240 PMC4026248

[B66] Koide F. Goebel S. Snyder B. Walters K. B. Gast A. Hagelin K.. (2016). Development of a Zika virus infection model in cynomolgus macaques. Front. Microbiol. 7:2028. 10.3389/fmicb.2016.02028 28066354 PMC5165249

[B67] Kruger R. P. (2016). Zika virus on the move. Cell 164, 585–587. 10.1016/j.cell.2016.01.040

[B68] Lamb L. E. Bartolone S. N. Kutluay S. B. Robledo D. Porras A. Plata M.. (2016). Advantage of urine based molecular diagnosis of Zika virus. Int. Urol. Nephrol. 48, 1961–1966. 10.1007/s11255-016-1406-9 27567913

[B69] Lambe U. Prasad M. Brar B. Guray M. Ikbal Ranjan K. (2016). Nanodiagnostics: a new frontier for veterinary and medical sciences. J. Exp. Biol. Agric. Sci. 4.307–320. 10.18006/2016.4(3S).307.320

[B70] Lanciotti R. S. Kosoy O. L. Laven J. J. Velez J. O. Lambert A. J. Johnson A. J.. (2008). Genetic and serologic properties of Zika virus associated with an epidemic, Yap state, Micronesia, 2007. Emerg. Infect. Dis. 14, 1232–1239. 10.3201/eid1408.080287 18680646 PMC2600394

[B71] Landry M. L. St. George K. (2017). Laboratory diagnosis of Zika virus infection. Arch. Pathol. Lab. Med. 141, 60–67. 10.5858/arpa.2016-0406-SA 27763787

[B72] Lazear H. M. Diamond M. S. (2016). Zika virus: new clinical syndromes and its emergence in the Western Hemisphere. J. Virol. 90, 4864–4875 10.1128/JVI.00252-16 26962217 PMC4859708

[B73] Lee D. Shin Y. Chung S. Hwang K. S. Yoon D. S. Lee J. H. (2016). Simple and highly sensitive molecular diagnosis of Zika virus by lateral flow assays. Anal. Chem. 88, 12272–12278. 10.1021/acs.analchem.6b03460 28193014

[B74] Leung G. H. Baird R. W. Druce J. Anstey N. M. (2015). Zika virus infection in Australia following a monkey bite in Indonesia. Southeast Asian J. Trop. Med. Public Health. 46, 460–464. 26521519

[B75] L'Huillier A. G. Hamid-Allie A. Kristjanson E. Papageorgiou L. Hung S. Wong C. F.. (2017a). Evaluation of Euroimmun anti-Zika virus IgM and IgG enzyme-linked immunosorbent assays for Zika virus serologic testing. J. Clin. Microbiol. 55, 2462–2471. 10.1128/JCM.00442-17 28566316 PMC5527425

[B76] L'Huillier A. G. Lombos E. Tang E. Perusini S. Eshaghi A. Nagra S. (2017b). Evaluation of altona diagnostics realstar Zika virus RT-PCR test kit for Zika virus PCR testing. J. Clin. Microbiol. 55, 1576–1584. 10.1128/JCM.02153-16 28298448 PMC5405276

[B77] Lum F. M. Lin C. Susova O. Y. Teo T. H. Fong S. W. Mak T. M. (2017). Sensitive detection of Zika virus antigen in patients' whole blood as an alternative diagnostic approach. J. Infect. Dis. 216, 182–190. 10.1093/infdis/jix276 28586426 PMC5853302

[B78] Lustig Y. Zelena H. Venturi G. Van Esbroeck M. Rothe C. Perret C. (2017). Sensitivity and kinetics of a NS1-based Zika virus ELISA in Zika infected travelers from Israel, Czech Republic, Italy, Belgium, Germany and Chile. J. Clin. Microbiol. 55, 1894–1901. 10.1128/JCM.00346-17 28381608 PMC5442546

[B79] Marchette N. J. Garcia R. Rudnick A. (1969). Isolation of Zika virus from *Aedes aegypti* mosquitoes in Malaysia. Am. J. Trop. Med. Hyg. 18, 411–415. 10.4269/ajtmh.1969.18.411 4976739

[B80] Martines R. B. Bhatnagar J. Keating M. K. Silva-Flannery L. Muehlenbachs A. Gary J.. (2016). Notes from the Field: evidence of Zika virus infection in brain and placental tissues from two congenitally infected newborns and two fetal losses - Brazil, 2015. MMWR Morb. Mortal. Wkly. Rep. 65, 159–160. 10.15585/mmwr.mm6506e1 26890059

[B81] Metsky H. C. Matranga C. B. Wohl S. Schaffner S. F. Freije C. A. Winnicki S. M.. (2017). Zika virus evolution and spread in the Americas. Nature 546, 411–415. 10.1038/nature22402 28538734 PMC5563848

[B82] Miller E. Becker Z. Shalev D. Lee C. T. Cioroiu C. Thakur K. (2017). Probable Zika virus-associated Guillain-Barré syndrome: challenges with clinico-laboratory diagnosis. J. Neurol. Sci. 375, 367–370. 10.1016/j.jns.2017.02.029 28320169

[B83] Mlakar J. Korva M. Tul N. Popović M. Poljšak-Prijatelj M. Mraz J.. (2016). Zika virus associated with microcephaly. N. Engl. J. Med. 374, 951–958. 10.1056/NEJMoa1600651 26862926

[B84] Monath T. P. Craven R. B. Muth D. J. Trautt C. J. Calisher C. H. Fitzgerald S. A. (1980). Limitations of the complement-fixation test for distinguishing naturally acquired from vaccine induced yellow fever infection in flavivirus-hyperendemic areas. Am. J. Trop. Med. Hyg. 29, 624–634. 10.4269/ajtmh.1980.29.624 7406113

[B85] Morrissey J. Jiang Q. Chandar Y. Cao S. Kharasch E. Singamaneni S. (2017). Rapid, point-of-care, thermally stable paper-based plasmonic assay for Zika virus diagnosis, in Advanced Photonics 2017 (IPR, NOMA, Sensors, Networks, SPPCom, PS), OSA Technical Digest (online) (Optical Society of America, 2017), paper SeTu1E.2.

[B86] Moulin E. Selby K. Cherpillod P. Kaiser L. Boillat-Blanco N. (2016). Simultaneous outbreaks of dengue, chikungunya and Zika virus infections: diagnosis challenge in a returning traveller with nonspecific febrile illness. New Microbes New Infect. 11, 6–7. 10.1016/j.nmni.2016.02.003 27006779 PMC4786754

[B87] Müller J. A. Harms M. Schubert A. Mayer B. Jansen S. Herbeuval J. P.. (2017). Development of a high-throughput colorimetric Zika virus infection assay. Med. Microbiol. Immunol. 206, 175–185. 10.1007/s00430-017-0493-2 28176006 PMC5357303

[B88] Munjal A. Khandia R. Dhama K. Sachan S. Karthik K. Tiwari R.. (2017a). Advances in developing therapies to combat Zika virus: current knowledge and future perspectives. Front. Microbiol. 8:1469. 10.3389/fmicb.2017.01469 28824594 PMC5541032

[B89] Munjal A. Khandia R. Tiwari R. Chakraborty S. Karthik K. Dhama K. (2017b). Advances in designing and developing vaccines against Zika virus. Int. J. Pharmacol. 13, 667–676. 10.3923/ijp.2017.667.676

[B90] Murray K. O. Gorchakov R. Carlson A. R. Berry R. Lai L. Natrajan M.. (2017). Prolonged detection of zika virus in vaginal secretions and whole blood. Emerg. Infect. Dis. 23, 99–101. 10.3201/eid2301.161394 27748649 PMC5176245

[B91] Musso D. Gubler D. J. (2016). Zika virus. Clin. Microbiol. Rev. 29, 487–524. 10.1128/CMR.00072-15 27029595 PMC4861986

[B92] Musso D. Roche C. Nhan T. X. Robin E. Teissier A. Cao-Lormeau VM. (2015). Detection of Zika virus in saliva. J. Clin. Virol. 68, 53–55. 10.1016/j.jcv.2015.04.021 26071336

[B93] Nicolini A. M. McCracken K. E. Yoon J. Y. (2017). Future developments in biosensors for field-ready Zika virus diagnostics. J. Biol. Eng. 11:7. 10.1186/s13036-016-0046-z 28127399 PMC5260080

[B94] Nikolay A. Castilho L. R. Reichl U. Genzel Y. (in press). Propagation of Brazilian Zika virus strains in static suspension cultures using Vero BHK cells. Vaccine. 10.1016/j.vaccine.2017.03.018 28343780

[B95] Nishiura H. Kinoshita R. Mizumoto K. Yasuda Y. Nah K. (2016). Equation Title: transmission potential of Zika virus infection in the South Pacific. Int. J. Infect. Dis. 45, 95–97. 10.1016/j.ijid.2016.02.017 26923081

[B96] Novotny J. Svobodova J. Ransnas L. A. Kubistova K. (1992). A method for the preparation of purified antigens of coxsackie virus B3 from a large volume of cell culture supernatant. Acta Virol. 36, 483–487. 1364026

[B97] Oliveira Melo A. S. Malinger G. Ximenes R. Szejnfeld P. O. Alves Sampaio S. Bispo de Filippis A. M. (2016). Zika virus intrauterine infection causes fetal brain abnormality and microcephaly: tip of the iceberg? Ultrasound Obstet. Gynecol. 47, 6–7. 10.1002/uog.15831 26731034

[B98] Pardee K. Green A. A. Takahashi M. K. Braff D. Lambert G. Lee J. W.. (2016). Rapid, low cost detection of Zika virus using programmable biomolecular components. Cell 165, 1255–1266. 10.1016/j.cell.2016.04.059 27160350

[B99] Pardy R. D. Rajah M. M. Condotta S. A. Taylor N. G. Sagan S. M. Richer M. J. (2017). Analysis of the T Cell response to Zika virus and identification of a novel CD8+ T cell epitope in immunocompetent mice. PLoS Pathog. 13:e1006184. 10.1371/journal.ppat.1006184 28231312 PMC5322871

[B100] Parra-Saavedra M. Reefhuis J. Piraquive J. P. Gilboa S. M. Badell M. L.. (2017). Serial head and brain imaging of 17 fetuses with confirmed Zika virus infection in Colombia, South America. Obstet. Gynecol. 130, 207–212. 10.1097/AOG.0000000000002105 28594771 PMC5511628

[B101] Pasquier C. Joguet G. Mengelle C. Chapuy-Regaud S. Pavili L. Prisant N.. (2018). Kinetics of anti-ZIKV antibodies after Zika infection using two commercial enzyme-linked immunoassays. Diagn. Microbiol. Infect. Dis. 90, 26–30. 10.1016/j.diagmicrobio.2017.09.001 29107414

[B102] Pessôa R. Patriota J. V. de Souza Mde L. Abd El Wahed A. Sanabani S. S. (2016). Detection of Zika *virus* in Brazilian patients during the first five days of infection - urine versus plasma. Euro Surveill. 21:30302. 10.2807/1560-7917.ES.2016.21.30.30302 27494130

[B103] Petersen L. R. Jamieson D. J. Powers A. M. Honein M. A. (2016). Zika virus. N. Engl. J. Med. 374, 1552–1563. 10.1056/NEJMra1602113 27028561

[B104] Pinto V. L. Jr. Luz K. Parreira R. Ferrinho P. (2015). Zika virus: a review to clinicians. Acta Med. Port. 28, 760–765. 26849762

[B105] Priye A. Bird S. W. Light Y. K. Ball C. S. Negrete O. A. Meagher R. J. (2017). A smart phone-based diagnostic platform for rapid detection of Zika, chikungunya, and dengue viruses. Sci. Rep. 7:44778 10.1038/srep44778 28317856 PMC5357913

[B106] Pyzocha N. J. Chinchen S. E. Maurer D. M. (2017). Zany over Zika virus: an overview of diagnosis and treatment modalities. Curr. Sports Med. Rep. 16, 109–113. 10.1249/JSR.0000000000000346 28282358

[B107] Quick J. Grubaugh N. D. Pullan S. T. Claro I. M. Smith A. D. Gangavarapu K.. (2017). Multiplex PCR method for MinION and Illumina sequencing of Zika and other virus genomes directly from clinical samples. Nat. Protoc. 12, 1261–1276. 10.1038/nprot.2017.066 28538739 PMC5902022

[B108] Rafiei N. Hajkowicz K. Redmond A. Taylor C. (2016). First report of Zika virus infection in a returned traveller from the Solomon Islands. Med. J. Aust. 204, 186. 10.5694/mja15.01275 26985845

[B109] Ratcliff R. M. Chang G. Kok T. Sloots T. P. (2007). Molecular diagnosis of medical viruses. Curr Iss Molr Biol. 9, 87–102. 17489437

[B110] Rather I. A. Kumar S. Bajpai V. K. Lim J. Park YH. (2017). Prevention and control strategies to counter Zika epidemic. Front. Microbiol. 8:305. 10.3389/fmicb.2017.00305 28293228 PMC5328966

[B111] Ren P. Ortiz D. A. Terzian A. C. B. Colombo T. E. Nogueira M. L. Vasilakis N.. (2017). Evaluation of Aptima Zika virus assay. J. Clin. Microbiol. 55, 2198–2203. 10.1128/JCM.00603-17 28468854 PMC5483922

[B112] Rodriguez-Morales A. J. Galindo-Marquez M. L. García-Loaiza C. J. Sabogal-Roman J. A. Marin-Loaiza S. Ayala A. F.. (2016). Mapping Zika virus infection using geographical information systems in Tolima, Colombia, 2015-2016. F1000Res. 5:568. 10.12688/f1000research.8436.1 27134732 PMC4837980

[B113] Rodriguez-Morales A. J. Galindo-Marquez M. L. García-Loaiza C. J. Sabogal-Roman J. A. Marin-Loaiza S. Ayala A. F. Lagos-Grisales G. J.. (2017a). Mapping Zika virus disease incidence in Valle del Cauca. Infection 45, 93–102. 10.1007/s15010-016-0948-1 27743307

[B114] Rodriguez-Morales A. J. Ruiz P. Tabares J. Ossa C. A. Yepes-Echeverry M. C. Ramirez-Jaramillo V.. (2017b). Mapping the ecoepidemiology of Zika virus infection in urban and rural areas of Pereira, Risaralda, Colombia, 2015-2016: implications for public health and travel medicine. Travel Med. Infect. Dis. 18, 57–66. 10.1016/j.tmaid.2017.05.004 28487212

[B115] Rossi S. L. Tesh R. B. Azar S. R. Muruato A. E. Hanley K. A. Auguste A. J.. (2016). Characterization of a novel murine model to study Zika virus. Am. J. Trop. Med. Hyg. 94, 1362–1369. 10.4269/ajtmh.16-0111 27022155 PMC4889758

[B116] Rossini G. Gaibani P. Vocale C. Cagarelli R. Landini M. P. (2017). Comparison of Zika virus (ZIKV) RNA detection in plasma, whole blood and urine - case series of travel-associated ZIKV infection imported to Italy, 2016. J. Infect. 75, 242–245. 10.1016/j.jinf.2017.05.021 28648495

[B117] Rozé B. Najioullah F. Fergé J. L. Apetse K. Brouste Y. Cesaire R.. (2016). Zika virus detection in urine from patients with Guillain-Barré syndrome on Martinique. Euro Surveill. 21:30154. 10.2807/1560-7917.ES.2016.21.9.30154 26967758

[B118] Sakudo A. Viswan A. Chou H. Sasaki T. Ikuta K. Nagatsu M. (2016). Capture of Dengue viruses using antibody-integrated graphite-encapsulated magnetic beads produced using gas plasma technology. Mol. Med. Rep. 14, 697–704. 10.3892/mmr.2016.5330 27221214 PMC4918612

[B119] Sánchez-Purrà M. Carré-Camps M. de Puig H. Bosch I. Gehrke L. Hamad-Schifferli K. (2017). Surface-enhanced Raman spectroscopy based sandwich immunoassays for multiplexed detection of zika and dengue viral biomarkers. ACS Infect Dis. 3, 767–776. 10.1021/acsinfecdis.7b00110. 28875696 PMC11323068

[B120] Saraswati T. E. Ogino A. Nagatsu M. (2012). Plasma-activated immobilization of biomolecules on to graphite-encapsulated magnetic nanoparticles. Carbon N.Y. 50, 1253–1261. 10.1016/j.carbon.2011.10.044

[B121] Shadgan B. Pakravan A. Zaeimkohan H. Shahpar F. M. Khodaee M. (2016). Zika and Rio Olympic games. Curr. Sports Med. Rep. 15, 298–300. 10.1249/JSR.0000000000000278 27399828

[B122] Shan C. Ortiz D. A. Yang Y. Wong S. J. Kramer L. D. Shi P.-Y.. (2017b). Evaluation of a novel reporter virus neutralization test for the serological diagnosis of Zika and dengue virus infection. J. Clin. Microbiol. 10.1128/JCM.00975-17 28768729 PMC5625389

[B123] Shan C. Xie X. Ren P. Loeffelholz M. J. Yang Y. Furuya A.. (2017a). A rapid Zika diagnostic assay to measure neutralizing antibodies in patients. EBioMedicine 17, 157–162. 10.1016/j.ebiom.2017.03.006 28283425 PMC5360589

[B124] Shankar A. Patil A. A. Skariyachan S. (2017). Recent perspectives on genome, transmission, clinical manifestation, diagnosis, therapeutic strategies, vaccine developments, and challenges of Zika virus research. Front. Microbiol. 8:1761. 10.3389/fmicb.2017.01761 28959246 PMC5603822

[B125] Sharma A. Lal S. K. (2017). Zika virus: transmission, detection, control, and prevention. Front. Microbiol. 8:110. 10.3389/fmicb.2017.00110 28217114 PMC5290000

[B126] Shinohara K. Kutsuna S. Takasaki T. Moi M. L. Ikeda M. Kotaki A.. (2016). Zika fever imported from Thailand to Japan, and diagnosed by PCR in the urines. J. Travel. Med. 23:tav011. 10.1093/jtm/tav011 26782128

[B127] Shukla S. Hong S. Y. Chung S. H. Kim M. (2016). Rapid detection strategies for the global threat of Zika virus: current state, new hypotheses, and limitations. Front. Microbiol. 7:1685. 10.3389/fmicb.2016.01685 27822207 PMC5075579

[B128] Singh R. K. Badasara S. K. Dhama K. Malik Y. P. S. (2015). Progress and prospects in vaccine research, in National Workshop on “Current Trends and Future Research Challenges in Vaccines and Adjuvants” (Bareilly: Organized at ICAR Indian Veterinary Research Institute), 1–19.

[B129] Singh R. K. Dhama K. Malik Y. S. Ramakrishnan M. A. Karthik K. Khandia R.. (2017). Ebola virus – epidemiology, diagnosis and control: threat to humans, lessons learnt, and preparedness plans- an update on its 40 year's journey. Vet. Quart 37, 98–135. 10.1080/01652176.2017.1309474 28317453

[B130] Singh R. K. Dhama K. Malik Y. S. Ramakrishnan M. A. Karthik K. Tiwari R.. (2016). Zika virus – emergence, evolution, pathology, diagnosis, and control: current global scenario and future perspectives – a comprehensive review. Vet. Quart. 36, 150–175. 10.1080/01652176.2016.1188333 27158761

[B131] Society for Maternal-Fetal Medicine (SMFM) Publications Committee (2016). Ultrasound screening for fetal microcephaly following Zika virus exposure. Am. J. Obstet. Gynecol. 214, B2–4. 10.1016/j.ajog.2016.02.043 26901275

[B132] Song J. Mauk M. G. Hackett B. A. Cherry S. Bau H. H. Liu C. (2016). Instrument-free point-of-care molecular detection of Zika virus. Anal. Chem. 88, 7289–7294. 10.1021/acs.analchem.6b01632 27306491 PMC4955015

[B133] Staples J. E. Dziuban E. J. Fischer M. Cragan J. D. Rasmussen S. A. Cannon M. J.. (2016). Interim guidelines for the evaluation and testing of infants with possible congenital Zika virus infection United States. MMWR Morb. Mortal. Wkly. Rep. 65, 63–67. 10.15585/mmwr.mm6503e3 26820387

[B134] Steinhagen K. Probst C. Radzimski C. Schmidt-Chanasit J. Emmerich P. van Esbroeck M.. (2016). Serodiagnosis of Zika virus (ZIKV) infections by a novel NS1-based ELISA devoid of cross-reactivity with dengue virus antibodies: a multicohort study of assay performance, 2015 to 2016. Euro Surveill. 21:30426. 10.2807/1560-7917.ES.2016.21.50.30426 28006649 PMC5291135

[B135] St. George K. Sohi I. S. Dufort E. M. Dean A. B. White J. L. Limberger R.. (2017). Zika virus testing considerations: lessons learned from the first 80 real-time reverse transcription-PCR-positive cases diagnosed in New York State. J. Clin. Microbiol. 55, 535–544. 10.1128/JCM.01232-16 27927917 PMC5277524

[B136] Sumita L. M. Rodrigues J. P. Ferreira N. E. Felix A. C. Souza N. C. Machado C. M.. (2016). Detection of human anti-zika virus IgG by Elisa using an antigen from *in vitro* infected Vero cells: preliminary results. Rev. Inst. Med. Trop. Sao Paulo. 58:89. 10.1590/s1678-9946201658089 27982355 PMC5147719

[B137] Tan S. K. Sahoo M. K. Milligan S. Taylor N. Pinsky B. A. (2017). Stability of Zika virus in urine: specimen processing considerations and implications for the detection of RNA targets in urine. J. Virol. Methods. 248, 66–70. 10.1016/j.jviromet.2017.04.018. 28472623

[B138] Tian B. Qiu Z. Ma J. Zardán Gómez de la Torre T. Johansson C. Svedlindh P.. (2016). Attomolar Zika virus oligonucleotide detection based on loop-mediated isothermal amplification and AC susceptometry. Biosens. Bioelectron. 86, 420–425. 10.1016/j.bios.2016.06.085 27423039

[B139] Tognarelli J. Ulloa S. Villagra E. Lagos J. Aguayo C. Fasce R.. (2015). A report on the outbreak of Zika virus on Easter Island, South Pacific, 2014. Arch. Virol. 161, 665–668. 10.1007/s00705-015-2695-5 26611910

[B140] Troncoso A. (2016). Zika threatens to become a huge worldwide pandemic. Asian Pac. J. Trop. Biomed. 6, 520–527. 10.1016/j.apjtb.2016.04.004

[B141] Van den Hurk R. Evoy S. (2015). A review of membrane-based biosensors for pathogen detection. Sensors. 15, 14045–14078. 10.3390/s150614045 26083229 PMC4507637

[B142] Waggoner J. J. Pinsky B. A. (2016). Zika virus: diagnostics for an emerging pandemic threat. J. Clin. Microbiol. 54, 860–867. 10.1128/JCM.00279-16 26888897 PMC4809954

[B143] Wang X. Yin F. Bi Y. Cheng G. Li J. Hou L.. (2016). Rapid and sensitive detection of Zika virus by reverse transcription loop-mediated isothermal amplification. J. Virol. Methods. 238, 86–93. 10.1016/j.jviromet.2016.10.010 27793644

[B144] Way J. H. Bowen E. T. Platt G. S. (1976). Comparative studies of some African arboviruses in cell culture and in mice. J. Gen. Virol. 30, 123–130. 10.1099/0022-1317-30-1-123 1245842

[B145] Weaver S. C. Costa F. Garcia-Blanco M. A. Ko A. I. Ribeiro G. S. Saade G.. (2016). Zika virus: history, emergence, biology, and prospects for control. Antiviral Res. 130, 69–80. 10.1016/j.antiviral.2016.03.010 26996139 PMC4851879

[B146] Wilson H. L. Tran T. Druce J. Dupont-Rouzeyrol M. Catton M. (2017). Neutralization assay for zika and dengue viruses by use of real-time-PCR-based end point assessment. J. Clin. Microbiol. 55, 3104–3112. 10.1128/JCM.00673-17 28794181 PMC5625395

[B147] Wong S. J. Furuya A. Zou J. Xie X. Dupuis A. P. Kramer L. D.. (2017). A multiplex microsphere immunoassay for Zika virus diagnosis. EBioMedicine 16, 136–140. 10.1016/j.ebiom.2017.01.008 28094237 PMC5474433

[B148] Xu M. Y. Liu S. Q. Deng C. L. Zhang Q. Y. Zhang B. (2016). Detection of Zika virus by SYBR green one-step real-time RT-PCR. J. Virol. Methods. 236, 93–97. 10.1016/j.jviromet.2016.07.014 27444120

[B149] Yadav S. Rawal G. Baxi M. (2016). Zika virus: a pandemic in progress. J. Transl. Int. Med. 4, 42–45. 10.1515/jtim-2016-0009 28191517 PMC5290914

[B150] Yang Y. Wong G. Ye B. Li S. Li S. Zheng H.. (2017). Development of a reverse transcription quantitative polymerase chain reaction-based assay for broad coverage detection of African and Asian Zika virus lineages. Virol. Sin. 32, 199–206. 10.1007/s12250-017-3958-y 28530022 PMC6598927

[B151] Yaren O. Alto B. W. Gangodkar P. V. Ranade S. R. Patil K. N. Bradley K. M.. (2017). Point of sampling detection of Zika virus within a multiplexed kit capable of detecting dengue and chikungunya. BMC Infect. Dis. 17:293. 10.1186/s12879-017-2382-0 28427352 PMC5399334

[B152] Zaghloul H. El-shahat M. (2014). Recombinase polymerase amplification as a promising tool in hepatitis C virus diagnosis. World J. Hepatol. 6, 916–922. 10.4254/wjh.v6.i12.916 25544878 PMC4269910

[B153] Zanluca C. de Melo V. C. Mosimann A. L. Dos Santos G. I. Dos Santos C. N. Luz K. (2015). First report of autochthonous transmission of Zika virus in Brazil. Mem. Inst. Oswaldo Cruz 110, 569–572. 10.1590/0074-02760150192 26061233 PMC4501423

[B154] Zanluca C. Dos Santos C. N. (2016). Zika virus an overview. Microbes Infect. 18, 295–301. 10.1016/j.micinf.2016.03.003 26993028

[B155] Zare Mehrjardi M. Keshavarz E. Poretti A. Hazin A. N. (2016). Neuroimaging findings of Zika virus infection: a review article. Jpn. J. Radiol. 34, 765–770. 10.1007/s11604-016-0588-5 27714487

[B156] Zare Mehrjardi M. Poretti A. Huisman T. A. Werner H. Keshavarz E. Araujo Júnior E. (2017). Neuroimaging findings of congenital Zika virus infection: a pictorial essay. Jpn. J. Radiol. 35, 89–94. 10.1007/s11604-016-0609-4 28074379

[B157] Zaytseva N. V. Montagna R. A. Lee E. M. Baeumner A. J. (2004). Multi-analyte single-membrane biosensor for the serotype-specific detection of Dengue virus. Anal. Bioanal. Chem. 380, 46–53. 10.1007/s00216-004-2724-9 15365670

[B158] Zhang B. Pinsky B. A. Ananta J. S. Zhao S. Arulkumar S. Wan H.. (2017). Diagnosis of Zika virus infection on a nanotechnology platform. Nat. Med. 23, 548–550. 10.1038/nm.4302 28263312

